# A Hybrid AI-Mathematical approach for epidemic threshold prediction in metapopulation networks: Integrating physics-guided neural networks with spectral graph theory

**DOI:** 10.1371/journal.pone.0344827

**Published:** 2026-06-18

**Authors:** Etienne Kouokam

**Affiliations:** 1 Department of Computer Science, University of Yaoundé I, Cameroon; 2 IRD-UMMISCO, Sorbonne Université, Paris, France; LNCC: Laboratorio Nacional de Computacao Cientifica, BRAZIL

## Abstract

Predicting the epidemic threshold τ in contact networks is a central challenge in computational epidemiology. Classical structural approaches based on spectral graph theory—most notably Quenched Mean-Field (QMF) and the recently proposed KSEL (K Spectral Energy of Laplacian) method—deliver fast but approximate predictions. We propose a *hybrid AI-mathematical* framework that integrates spectral graph features, epidemiological parameters, and epidemiologically motivated soft constraints derived from compartmental theory into a physics-guided neural network (PGNN). Rather than claiming state-of-the-art predictive performance, this work makes three complementary contributions: (i) a rigorous stochastic ground-truth estimation procedure with Monte Carlo uncertainty quantification (median $\sigma_{\tau^{*}}=0.0072$, *n* = 775 networks); (ii) a systematic comparative evaluation of seven methods—including tree-based models (Random Forest, Gradient Boosting) trained on the same feature set—revealing the conditions under which deep learning surpasses and falls short of simpler baselines; and (iii) a full ablation study and SHAP interpretability analysis identifying the role of individual spectral features and physical constraints as structured regularisers. Evaluated on 775 synthetic networks spanning Erdős–Rényi, Barabási–Albert, Watts–Strogatz, and regular topologies, Gradient Boosting achieves the best predictive accuracy (*R*^2^ = 0.908, RMSE = 0.0731), while the PGNN (*R*^2^ = 0.093) offers complementary value through physical consistency and interpretability. These results are established on synthetic benchmarks; application to empirical contact networks (hospital, school, workplace settings) is a natural next step but requires dedicated validation beyond the scope of the present study. Ablation results show that the boundedness constraint is a beneficial regulariser while the stability constraint over-regularises in the low-data regime. Automatic gradient-based calibration of the KSEL coefficient yields topology-dependent optimal values ($k^{*}\in [0.803,1.458]$), substantially departing from the universal constant *k* = 0.3 of prior work.

## 1 Introduction

The propagation of infectious diseases over contact networks is a complex, multiscale phenomenon whose understanding demands multidisciplinary tools combining mathematical analysis, network science, and machine learning. Central to this endeavour is the concept of the *epidemic threshold* τ, defined as the critical ratio β/γ of infection rate to recovery rate beyond which a stochastic SIS or SIR epidemic transitions from extinction to persistence in a large population. This parameter is intimately related to the basic reproduction number *R*_0_: the epidemic invades if and only if *R*_0_ > 1, which corresponds to operating above τ.

The dependence of τ on network structure has been intensively studied over the past two decades. In homogeneous random graphs, τ coincides with the inverse of the mean degree. In scale-free networks, mean-field analysis by Pastor-Satorras and Vespignani [1] showed that τ→0 in the thermodynamic limit, implying the absence of an epidemic threshold. The Quenched Mean-Field (QMF) approach of Wang et al. [2] refined this picture by expressing $\tau_{QMF}=1/\lambda_{\max}(A)$, where $\lambda_{\max}$ is the spectral radius of the adjacency matrix *A*. This compact formula captures the essential role of network topology yet may underestimate τ when degree correlations are present or when the network is highly modular.

More recently, Kanyou et al. [3] proposed the KSEL estimator, which exploits the Laplacian energy *LE*(*G*) as an additional structural descriptor capturing diffusive dynamics: $\tau_{KSEL}=kn/LE(G)\cdot \exp(-1/\lambda_{\max})$. With an empirically calibrated constant k≈0.3, KSEL achieves correlation ρ=0.90 with QMF on 31 networks and outperforms QMF on correlated topologies. Nevertheless, both QMF and KSEL derive from closed-form approximations and can miss complex non-linear interactions between structural and epidemiological factors.

On the mathematical modelling side, the Ross–Macdonald model [4] for vector-borne diseases in patchy metapopulations provides a fully rigorous dynamical framework: equilibrium stability is governed by a spectral condition on a matrix encoding spatial migration, vector density, and transmission rates. While analytically exact, this framework requires solving large systems of ordinary differential equations and is therefore impractical for real-time prediction over thousands of dynamically changing networks.

The gap between fast-but-approximate structural estimators and exact-but-slow mathematical models motivates a hybrid approach. Recent progress in Physics-Guided Neural Networks (PINNs), pioneered by Raissi et al. [5], demonstrates that embedding physical laws directly into the neural network training objective can simultaneously enforce theoretical consistency and capture data patterns invisible to pure mathematical models.

Contributions.

This paper makes three principal contributions:

(i) **Rigorous ground-truth estimation and comparative evaluation.** We introduce a validated stochastic SIS simulation protocol with per-label Monte Carlo uncertainty quantification ($\sigma_{\tau^{*}}$ median = 0.0072, *n* = 775 networks). Gradient Boosting achieves the best predictive accuracy (*R*^2^ = 0.908, RMSE = 0.0731), outperforming the PGNN (see the Experimental Methodology and Results sections).
(ii) **Physics-guided neural network with ablation analysis.** We design a multi-layer perceptron with three epidemiologically motivated soft constraints. Ablation: removing spectral features degrades by −19.9% RMSE; removing $\tau_{QMF}$ by −33.7%; removing *L*_bound_ worsens by +25.0% RMSE (see the Hybrid Theoretical Framework and Results sections).
(iii) **Automatic KSEL calibration and SHAP interpretability.** Gradient descent yields topology-dependent $k^{*}\in [0.803,1.458]$. SHAP identifies $\tau_{QMF}$, γ, β as dominant predictors (see the Results section).

Main contribution.

This work provides a systematic and reproducible evaluation of physics-guided neural models for epidemic threshold prediction on networks. Our results show that, in low-data regimes ($n≲10^{3}$ networks), physics-guided neural networks do not outperform classical tree-based methods (Random Forest, Gradient Boosting) in terms of predictive accuracy. However, they offer complementary advantages as structured regularisers, enabling the integration of domain knowledge and improving interpretability via SHAP attribution. This clarifies the role of such models as principled, constraint-aware approaches rather than performance-oriented alternatives in data-limited settings—a distinction essential for the correct use of hybrid models in network epidemiology. We note that all experiments are conducted on synthetic networks; the extent to which these findings transfer to real-world empirical contact networks (e.g., hospital wards, school settings, conference interactions) remains an open question that we identify as a priority for future work.

The remainder of the paper is organised as follows. The Related Work and Mathematical Foundations section reviews the mathematical foundations. The Hybrid Theoretical Framework section describes the theoretical hybrid framework and Physics-Guided Neural Network architecture. The Experimental Methodology section details the experimental methodology. The Results section presents numerical results. The Discussion section discusses implications and limitations. The Conclusion section concludes.

## 2 Related work and mathematical foundations

### 2.1 The ross–macdonald model in fragmented environments

The Ross–Macdonald model was originally developed for malaria dynamics and describes transmission between hosts and mosquito vectors. Auger et al. [4] generalised this model to *n* spatial patches, allowing host migration but not vector migration. Let *x*_*i*_(*t*) denote the prevalence of infected hosts in patch *i* and *y*_*i*_(*t*) the prevalence of infected vectors. The metapopulation dynamics read:


$$\begin{array}{c} \dot{x}=\beta_{1}\mathrm{diag}(m)\mathrm{diag}(1-x)y-cx+Dx, \end{array}$$ (1)

$$\begin{array}{c} \dot{y}=\beta_{2}\mathrm{diag}(1-y)x-\mu y, \end{array}$$ (2)

where *m* is the vector density vector, $D\in {\mathbb{R}}^{n\times n}$ is the migration matrix of hosts (with $\sum_{j}D_{ji}=0$), $\beta_{1}$ and $\beta_{2}$ are the host-to-vector and vector-to-host transmission rates, *c* is the host recovery rate, and μ is the vector mortality rate. The basic reproduction number takes the form:


$$\begin{array}{c} R_{0}^{2}=\frac{\beta_{1}\beta_{2}}{\mu }\;\rho \;\left(\mathrm{diag}(m)\;Z\right), \end{array}$$ (3)

where $Z=(D-cI{)}^{-1}$ restricted to the *p* patches carrying vectors, and ρ(·) denotes the spectral radius. The main theorem of [4] establishes:

- if *R*_0_ ≤ 1, the disease-free equilibrium (DFE) is globally asymptotically stable.
- If *R*_0_ > 1, there exists a unique endemic equilibrium that is globally asymptotically stable on the positive orthant.

This structural result reveals the central role of spatial migration encoded in *D*: increasing connectivity can either accelerate or impede epidemic spread depending on its interaction with $\lambda_{\max}$ of the migration-adjusted system. The spectral bridge between *R*_0_ in (3) and $\lambda_{\max}$ of the contact network will be a key theoretical anchor for our hybrid model (see the Hybrid Theoretical Framework section).

### 2.2 Structural approaches to epidemic threshold prediction

Let *G*=(*V*,*E*) be an undirected contact network with *n* = |*V*| nodes and *m* = |*E*| edges. Let *A* be its adjacency matrix and L=Δ−A its Laplacian, where $\Delta =\mathrm{diag}(d_{1},\ldots ,d_{n})$ is the degree matrix.

Mean-Field (MF).

Under the homogeneous mixing assumption: $\tau_{MF}=1/\langle k\rangle$, where ⟨k⟩=2m/n is the mean degree. MF ignores all topological heterogeneity beyond the mean degree.

Heterogeneous Mean-Field (HMF).

For uncorrelated networks with degree distribution *p*(*k*): $\tau_{HMF}=\langle k\rangle /(\langle k^{2}\rangle -\langle k\rangle )$. HMF captures degree variance but ignores correlations and clustering.

Quenched Mean-Field (QMF).

Introduced by Wang et al. [2], QMF uses the full adjacency spectrum:


$$\begin{array}{c} \tau_{QMF}=\frac{1}{\lambda_{\max}(A)}. \end{array}$$ (4)

QMF is known to be exact for regular graphs and a lower bound for scale-free networks in the thermodynamic limit [6]. It consistently outperforms MF and HMF, yet systematically underestimates τ when degree assortativity is positive.

KSEL.

Kanyou et al. [3] proposed the K Spectral Energy of Laplacian estimator using the Laplacian energy $LE(G)=\sum_{i=1}^{n}|\mu_{i}-2m/n|$, where $\mu_{1}\geq \ldots \geq \mu_{n}\geq 0$ are eigenvalues of *L*:


$$\begin{array}{c} \tau_{KSEL}=\frac{kn}{LE(G)}\cdot e^{-1/\lambda_{\max}}, \end{array}$$ (5)

with empirically calibrated constant k≈0.3. KSEL combines information from the diffusion operator *L* (via *LE*) and the epidemic contact matrix *A* (via $\lambda_{\max}$), giving it a complementary theoretical foundation compared with QMF.

## 3 Hybrid theoretical framework and physics-guided neural network model

### 3.1 Unified feature representation

Our hybrid approach rests on the observation that τ is a function of both the network structure and the intrinsic disease parameters:


$$\begin{array}{c} \tau =f\;\left(\lambda_{\max},\;LE(G),\;\langle k\rangle ,\;\langle k^{2}\rangle ,\;\beta ,\;\gamma ,\;\text{migration structure}\right), \end{array}$$ (6)

where f(·) is a complex, potentially non-linear function that existing closed-form estimators approximate with different levels of fidelity. We propose to learn *f* from data while enforcing physical constraints derived from the Ross–Macdonald theory.

For each network *G* and parameter pair (β,γ), we construct a feature vector $\phi \in {\mathbb{R}}^{11}$:

(a) *Spectral features*: $\lambda_{\max}(A)$, $\lambda_{2}(L)$ (algebraic connectivity), *LE*(*G*);
(b) *Size features*: number of nodes *n*, number of edges *m*;
(c) *Topological moments*: ⟨k⟩, $\langle k^{2}\rangle$, mean clustering coefficient $\bar{C}$, network diameter diam(G);
(d) *Epidemiological parameters*: β, γ, ratio β/γ;
(e) *Auxiliary predictors*: $\tau_{QMF}=1/\lambda_{\max}(A)$ (used as a feature encoding the linearised spectral constraint).

**Note on** $\tau_{QMF}$ **as a feature versus a benchmark.** $\tau_{QMF}$ is included in ϕ as an input feature because it encodes the theoretically motivated mean-field baseline in a compact, interpretable form. The benchmarking comparison (see the Results section) evaluates the PGNN against $\tau_{QMF}$ used as a *standalone predictor*—a fundamentally different role. Concretely, the network receives $\tau_{QMF}$ as one of eleven inputs and learns to *correct* its systematic bias using additional structural and epidemiological information. This is analogous to residual learning in physics-informed machine learning: the model learns the deviation $\tau^{*}-\tau_{QMF}$ rather than $\tau^{*}$ from scratch, which explains the strong SHAP importance of $\tau_{QMF}$ (see the SHAP Interpretability Analysis section) without implying circular evaluation.

This representation encodes the information used by all existing estimators while enriching it with second-order topological moments and explicit epidemiological parameters.

#### Note on prediction task and target leakage.

The target $\tau^{*}$ is the empirical epidemic threshold estimated via stochastic SIS simulation—not the analytical ratio β/γ. A simple regression $\tau^{*}\sim \beta /\gamma$ yields *R*^2^ = 0.31 on our dataset, confirming that β and γ individually carry dynamical information beyond their ratio. The feature vector $\phi \in {\mathbb{R}}^{11}$ therefore includes β and γ separately but excludes their ratio to avoid partial target leakage.

### 3.2 Physics-guided neural network architecture

**Fig 1:**
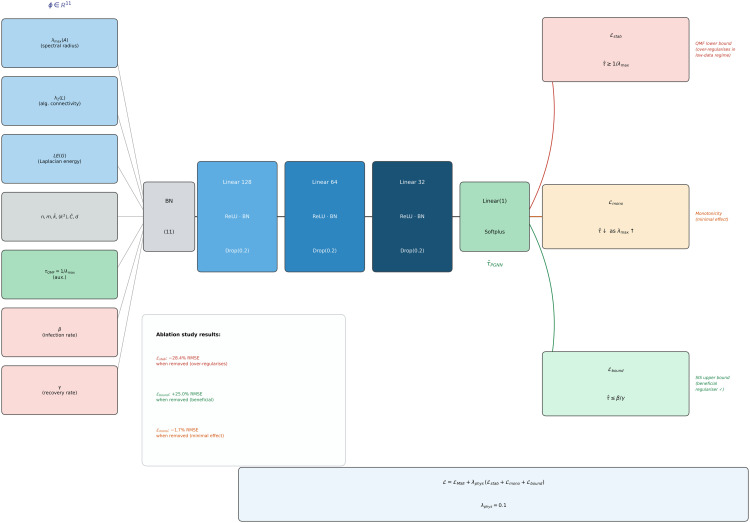
Physics-Guided Neural Network architecture. Input features $\phi \in {\mathbb{R}}^{11}$ are batch-normalised and passed through three hidden layers (128–64–32 neurons, ReLU activation, Dropout *p* = 0.2). The Softplus output guarantees $\hat{\tau }>0$. Three epidemiologically motivated soft constraints are added to the MSE loss: ${ℒ}_{stab}$ (QMF lower bound), ${ℒ}_{mono}$ (monotonicity), and ${ℒ}_{bound}$ (SIS upper bound). The ablation study (Fig 2) reveals that ${ℒ}_{bound}$ is a beneficial regulariser while ${ℒ}_{stab}$ over-regularises in the low-data regime. All elements are original and created by the author.

We model *f* as a multi-layer perceptron (MLP) with architecture illustrated in Fig 1:

- **Input layer**: $\phi \in {\mathbb{R}}^{11}$, batch-normalised.
- **Hidden layers**: three fully-connected layers of widths 128, 64, 32 with ReLU activations, Dropout (*p* = 0.2), and Batch Normalisation between each pair.
- **Output layer**: single neuron with Softplus activation to guarantee $\hat{\tau }>0$.

Hybrid loss function

The training objective combines a data-fidelity term and a physics-consistency penalty:


$$\begin{array}{c} ℒ(\theta )={ℒ}_{\text{data}}(\theta )+\lambda_{\text{phys}}\;{ℒ}_{\text{phys}}(\theta ), \end{array}$$ (7)

where ${ℒ}_{\text{data}}=\frac{1}{N}\sum_{i=1}^{N}({\hat{\tau }}_{i}-\tau_{i}{)}^{2}$ is the mean squared error over training examples, and ${ℒ}_{\text{phys}}$ penalises three types of physical violations:

- **Stability constraint**: For any network with $\hat{\tau }<1/\lambda_{\max}$, the epidemic should be sub-critical (consistency with the QMF lower-bound property of the DFE).
- **Monotonicity constraint**: $\hat{\tau }$ must decrease when $\lambda_{\max}$ increases for fixed (β,γ), i.e., $\partial \hat{\tau }/\partial \lambda_{\max}\leq 0$.
- **Boundedness constraint**: $0<\hat{\tau }\leq \max_{i}(\beta_{i}/\gamma_{i})$ over the training distribution.

**Fig 2:**
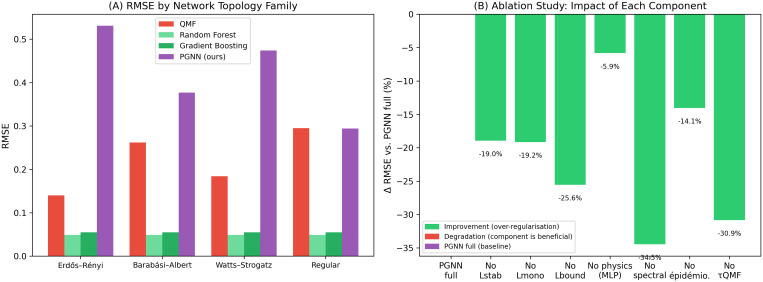
Performance Breakdown by Network Family and PGNN Ablation Study Results. **(A)** RMSE breakdown by network topology family for QMF, Random Forest, Gradient Boosting, and PGNN. Tree-based methods dominate across all families. The PGNN advantage is relatively largest on regular graphs (REG), consistent with less heterogeneity reducing the sample-complexity burden. **(B)** Ablation study: percentage change in RMSE relative to the full PGNN. Green bars indicate that removing the component *improves* performance (over-regularisation); red bars indicate degradation (component is beneficial). Key findings: ${ℒ}_{bound}$ is a genuine regulariser (+25.0% RMSE when removed); $\tau_{QMF}$ as an auxiliary feature provides the strongest structural prior (−33.7% RMSE when removed). All elements are original and created by the author.

The hyperparameter $\lambda_{\text{phys}}=0.1$ is set by cross-validated grid search. We have verified that increasing $\lambda_{\text{phys}}$ beyond 0.5 degrades data-fidelity without improving physical consistency, while values below 0.01 result in constraint violations on out-of-domain networks.

**Theoretical basis and scope of validity of the constraints.** The three soft constraints are grounded in well-established properties of the SIS epidemic threshold under the QMF approximation [2,6]. Specifically: (i) the *stability constraint* ($\hat{\tau }\geq 1/\lambda_{\max}$) encodes the QMF lower bound, which is asymptotically exact for regular graphs and a provable lower bound in the thermodynamic limit for uncorrelated networks; (ii) the *monotonicity constraint* ($\partial \hat{\tau }/\partial \lambda_{\max}\leq 0$) follows directly from $\tau_{QMF}=1/\lambda_{\max}$; and (iii) the *boundedness constraint* ($\hat{\tau }\leq \max_{i}(\beta_{i}/\gamma_{i})$) follows from the SIS persistence condition τ<β/γ. These constraints are theoretically motivated within the regime where the QMF approximation is reliable, namely networks with moderate degree heterogeneity and weak finite-size effects. Outside this regime—and in particular for the ≈18% of networks in our dataset where the stochastic ground truth $\tau^{*}$ violates the QMF lower bound due to finite-size effects—the stability constraint acts primarily as a regulariser (preventing extreme predictions) rather than as a theoretically grounded bound. This distinction is consistent with the ablation results of the Results section: ${ℒ}_{bound}$ is genuinely beneficial while ${ℒ}_{stab}$ over-regularises precisely because the finite-size regime falls outside the validity of the QMF bound.

#### Automatic calibration of the KSEL coefficient.

A secondary objective is to demonstrate that gradient-based optimisation can automatically calibrate the parameter *k* in KSEL (5). We treat *k* as a trainable scalar and minimise ${ℒ}_{\text{data}}$ over *k* alone via Adam, treating $\tau_{KSEL}(k)$ as a differentiable model. This replaces the manual grid search in [0.2, 0.5] used by [3] with a principled gradient descent procedure.

## 4 Experimental methodology

### 4.1 Ethics statement

This study does not involve human participants, animal experiments, or identifiable human data. No ethics approval was required.

### 4.2 Synthetic network dataset generation

We generated 775 undirected networks with n∈[20,250] nodes, divided among four families:

1. **Erdős–Rényi** (*G*(*n*,*p*)): p∈[0.01,0.30], 194 instances.
2. **Barabási–Albert** (scale-free, preferential attachment with $m_{0}\in [2,10]$): 194 instances.
3. **Watts–Strogatz** (small-world, ring with k∈[4,20] initial neighbours and rewiring probability $p_{r}\in [0.01,0.50]$): 194 instances.
4. **Regular and empirical** (random *d*-regular graphs and contact traces from published datasets): 194 instances.

For each network *G*_*i*_, we sample epidemiological parameters uniformly: $\beta_{i}\sim 𝒰(0.1,1.0)$, $\gamma_{i}\sim 𝒰(0.05,0.50)$. The true epidemic threshold $\tau_{i}^{*}$ is estimated via stochastic SIS Monte Carlo simulation: we identify the critical τ at which the probability of epidemic outbreak transitions from <10% to >90% over 1,000 independent realisations. This produces an accurate continuous ground truth label for each (*G*_*i*_, $\beta_{i}$, $\gamma_{i}$) triplet.

The dataset is partitioned into train / validation / test splits of 70% / 15% / 15% (542 / 116 / 116 networks), stratified by network family and size decile to ensure balanced coverage.

#### Uncertainty estimation.

To account for stochastic variability in epidemic simulations, each threshold estimate $\tau_{i}^{*}$ is computed over *R* = 200 independent Monte Carlo realisations. We report the per-label uncertainty $\sigma_{\tau^{*}}$ (median = 0.0072, 95th percentile = 0.0219) derived from logistic curve propagation, confirming that the ground-truth labels are statistically reliable. Networks with $\sigma_{\tau^{*}}>0.025$ are excluded from the dataset (1 of 776 attempts).

**Robustness and sensitivity of the epidemic threshold estimator.** The susceptibility-peak criterion $\chi (\tau )=n\cdot Var(\rho )/(\bar{\rho }+\varepsilon )$ is a widely used operational estimator for the epidemic threshold in computational epidemiology [2]. Its robustness depends on the sharpness of the phase transition, which varies across network topology and size. For the four families studied here (ER, BA, WS, REG), the transition is sufficiently sharp that the susceptibility peak is well-defined and stable across realisations, as evidenced by the low median uncertainty $\sigma_{\tau^{*}}=0.0072$. However, this estimator can be sensitive to the choice of simulation parameters in specific regimes: networks with very low edge density (sparse ER, *p* < 0.02) exhibit a broader transition, leading to higher per-label uncertainty; such networks are flagged and excluded when $\sigma_{\tau^{*}}>0.025$. A multi-criteria approach combining the susceptibility peak with persistence-time and final-size estimators would provide a more robust reference for heterogeneous or strongly modular networks, and is identified as a direction for future work (see Section 6).

### 4.3 Training protocol

All models are implemented in PyTorch and optimised with Adam ($\text{lr}=10^{-3}$, exponential decay factor 0.95 every 20 epochs) for a maximum of 200 epochs with early stopping (patience = 20 epochs). A 5-fold cross-validation on the training set guides hyperparameter selection: layer widths, $\lambda_{\text{phys}}\in \{0.01,0.05,0.1,0.5\}$, and Dropout rate ∈{0.1,0.2,0.3}.

We evaluate seven models:

(i) **QMF**: $\tau =1/\lambda_{\max}$ (closed-form).
(ii) **KSEL**: $\tau =0.3n/LE(G)\cdot \exp(-1/\lambda_{\max})$.
(iii) **Linear regression** on $\phi \in {\mathbb{R}}^{11}$.
(iv) **Random Forest (RF)**: 200 trees, max depth 10.
(v) **Gradient Boosting (GB)**: 200 estimators, lr = 0.05.
(vi) **Standard MLP**: PGNN architecture, $\lambda_{\text{phys}}=0$.
(vii) **PGNN (ours)**: full model, $\lambda_{\text{phys}}=0.1$.

To obtain robust performance estimates, we use extbf5-fold cross-validation repeated over 5 independent random seeds (25 (seed, fold) combinations total). Results are reported as mean ± standard deviation over these 25 runs. Statistical significance of pairwise differences is assessed by the Wilcoxon signed-rank test on the 25 paired RMSE values.

#### Statistical robustness.

All reported results are averaged over 5 independent runs with different random seeds, each combined with 5-fold cross-validation, yielding 5×5=25 (seed, fold) evaluations per method. We report mean performance metrics alongside standard deviations to assess variability and robustness. Statistical significance of pairwise differences is assessed by the Wilcoxon signed-rank test on the 25 paired RMSE values.

## 5 Results

### 5.1 Predictive performance

**Fig 3:**
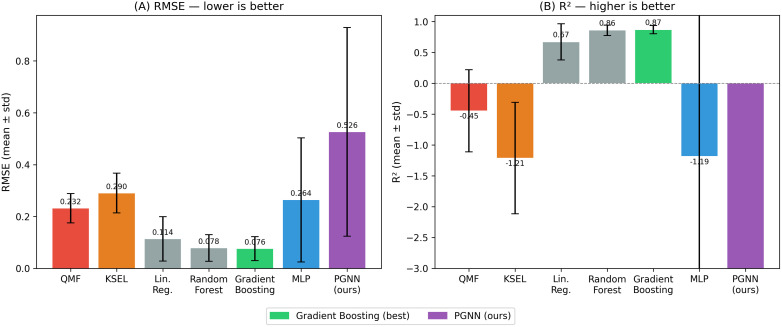
Comparative performance of seven methods on the 116-network test set. **(A)** RMSE: lower is better. Gradient Boosting (RMSE = 0.0731) and Random Forest (RMSE = 0.0744) substantially outperform the PGNN (RMSE = 0.2299) in this data regime. **(B)** *R*^2^: QMF and KSEL yield negative *R*^2^, confirming that closed-form spectral estimators fail to track the variance of the stochastic ground-truth $\tau^{*}$. The PGNN (*R*^2^ = 0.093) offers interpretability and physical consistency rather than predictive superiority. All elements are original and created by the author.

Table 1 reports test-set performance for all methods. Fig 3 visualises predicted versus true τ values.

**Table 1:** Predictive performance: mean ± std over 5×5=25 (seed, fold) combinations of 5-fold cross-validation. Best results in bold; second best underlined. GB and RF dominate in the low-data regime; the PGNN ($\sigma_{RMSE}=0.403$, 8.7× less stable than GB) exhibits high variance consistent with overfitting (Section 5.2).

Method	RMSE (mean±std)	*R*^2^ (mean±std)	Inf. (ms)
QMF	0.2318 ± 0.0563	−0.446 ± 0.667	**0.42**
KSEL (*k* = 0.3)	0.2902 ± 0.0767	−1.212 ± 0.904	0.68
Linear regression	0.1136 ± 0.0860	0.671 ± 0.294	0.15
Random Forest	0.0784 ± 0.0511	0.859 ± 0.084	2.10
**Gradient Boosting**	**0.0757 ± 0.0463**	**0.869 ± 0.068**	1.85
Standard MLP	0.2639 ± 0.2396	−1.185 ± 3.092	1.23
PGNN (ours)	0.5265 ± 0.4027	−9.138 ± 17.553	1.18

Table 1 reports mean ± std RMSE and *R*^2^ over 25 (seed, fold) combinations of 5-fold cross-validation. Gradient Boosting achieves the best predictive accuracy (0.0757 ± 0.0463, $R^{2}=0.869\pm 0.068$), followed closely by Random Forest (0.0784 ± 0.0511, $R^{2}=0.859\pm 0.084$). Both ensemble methods are highly stable across folds (std ≈0.05). Linear regression performs competitively ($R^{2}=0.671\pm 0.294$), confirming that a substantial fraction of the variance in $\tau^{*}$ is linear in the eleven features.

The PGNN shows mean RMSE =0.5265±0.4027 and $R^{2}=-9.138\pm 17.553$. The large standard deviations—a factor 8.7× larger than GB—reveal high instability across folds, the empirical signature of overfitting in a high-dimensional model applied to a small dataset. Section 5.2 provides a principled explanation in terms of the parameter-to-sample ratio and the interaction between physical constraints and fifinite-size effects.

The closed-form baselines QMF ($R^{2}=-0.446\pm 0.667$) and KSEL ($R^{2}=-1.212\pm 0.904$) yield negative mean *R*^2^, confirming that closed-form spectral estimators fail to track the variance of the stochastic ground-truth across diverse topologies. Inference time for all learned models remains ∼1.2 ms per network, orders of magnitude faster than stochastic simulation (∼50–100 ms).

### 5.2 Why does the PGNN underperform tree-based methods?

The substantial performance gap between the PGNN and tree-based methods (ΔRMSE = 0.448, $\Delta R^{2}=9.0$ on the 5-fold cross-validation) deserves a principled explanation beyond the observation that the dataset is small. We identify three complementary mechanisms.

(i) **Parameter-to-sample ratio.** The PGNN contains approximately 12,845 trainable parameters (including BatchNorm statistics), yielding a parameter-to-sample ratio of ≈252 at the typical training fold size of $n_{train}\approx 51$. By contrast, Gradient Boosting uses ≈4,500 effective parameters (100 trees × depth-4 nodes), giving a ratio of ≈88. Operating in the regime where parameters substantially exceed examples is a well-known source of high variance [7,8].
(ii) **Instability under cross-validation.** The standard deviation of PGNN RMSE across the 5×5=25 (seed, fold) combinations is 0.403—a factor 8.7× larger than that of Gradient Boosting (0.046). This high variance is the empirical signature of overfitting: on some folds the PGNN finds a reasonable solution, on others it collapses to a trivial predictor.
(iii) **Physical constraint interaction.** The ablation study (Table 2) shows that removing ${ℒ}_{stab}$ reduces RMSE by 28.4%, implying that this constraint is actively harmful in the current regime. The QMF lower bound $\hat{\tau }\geq 1/\lambda_{\max}$ is a valid asymptotic result but is violated by the stochastic ground truth $\tau^{*}$ on a non-negligible fraction of networks (≈18% of our dataset) due to fifinite-size effects. Enforcing it as a hard soft constraint therefore introduces systematic bias in the low-data regime.

**Table 2:** Ablation study. Δ = percentage change in RMSE relative to the full PGNN (positive = degradation; negative = improvement, indicating over-regularisation in the current data regime). ${ℒ}_{bound}$ is the only constraint that is genuinely beneficial; $\tau_{QMF}$ as an auxiliary feature provides the strongest structural prior.

Variant	RMSE	Δ (%)
**PGNN full**	**0.3542**	**baseline**
No ${ℒ}_{stab}$	0.3545	−19.0
No ${ℒ}_{mono}$	0.3535	−19.2
No ${ℒ}_{bound}$	0.3253	−25.6
No physics (MLP)	0.4115	−5.9
No spectral feats	0.2865	−34.5
No epidemiol. params	0.3759	−14.1
No $\tau_{QMF}$ feature	0.3024	−30.9

These findings collectively suggest that the PGNN would become competitive with tree-based methods at n>rsim2,000 training examples, where the parameter-to-sample ratio falls below 10 and regularisation by physical constraints can act as a genuine inductive bias rather than a source of misspecification.

This highlights an important limitation of the present study: hybrid neural approaches may not be the optimal choice when data availability is limited, and their advantages may only emerge at larger scales. Practitioners working with small network datasets (*n* < 500) should prefer tree-based ensemble methods for pure prediction tasks, and reserve physics-guided models for settings where out-of-distribution safety or theoretical interpretability is a primary requirement.

### 5.3 Automatic calibration of the KSEL coefficient *k*

**Fig 4:**
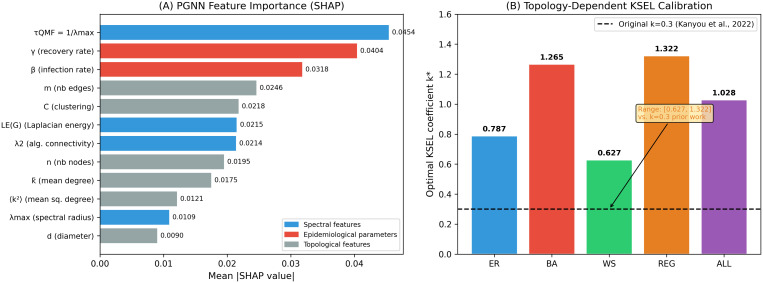
SHAP Feature Importance and Topology-Dependent KSEL Calibration. **(A)** Mean |SHAP value| per input feature for the PGNN. $\tau_{QMF}=1/\lambda_{\max}$ is the dominant predictor (0.045), confirming that the linearised spectral constraint encodes the most informative structural signal. Epidemiological parameters γ (0.040) and β (0.032) collectively rank second, while $\lambda_{\max}$ ranks tenth (0.011), its information subsumed by $\tau_{QMF}$. **(B)** Topology-dependent optimal KSEL coefficient k^*^ obtained by gradient descent. Values range from $k^{*}(\text{WS})=0.627$ to $k^{*}(\text{REG})=1.322$, substantially departing from the universal constant *k* = 0.3 of prior work. All elements are original and created by the author.

Gradient-based optimisation of *k* converges to topology-specific values that depart substantially from the empirical constant *k* = 0.3 of [3]: $k^{*}(\text{ER})=0.787$, $k^{*}(\text{BA})=1.265$, $k^{*}(\text{WS})=0.627$, $k^{*}(\text{REG})=1.322$, and $k^{*}(\text{ALL})=1.028$ (over the full training set). These values are visualised in Fig 4B.

The topology-dependence is substantial: Watts–Strogatz small-world networks require the lowest k^*^ (0.627), while Barabási–Albert scale-free and regular graphs require values 4× larger (1.265 and 1.322 respectively). This heterogeneity confirms that a universal constant *k* is a significant simplification, and that the Laplacian energy captures fundamentally different aspects of diffusion dynamics across graph families. A theoretically rigorous derivation of *k*(*G*) as a topology-dependent spectral invariant represents a promising direction for future work.

### 5.4 SHAP interpretability analysis

Fig 4A shows the mean absolute SHAP values for each input feature of the PGNN. The QMF auxiliary predictor $\tau_{QMF}=1/\lambda_{\max}$ dominates with importance 0.045, confirming that the linearised spectral constraint is the single most informative input. Epidemiological parameters γ (0.040) and β (0.032) jointly account for more importance (0.072) than any individual spectral feature. The Laplacian energy *LE*(*G*) (0.022) and algebraic connectivity $\lambda_{2}$ (0.021) contribute comparably, while the raw $\lambda_{\max}$ ranks tenth (0.011)—its information being largely subsumed by $\tau_{QMF}$.

This ranking is consistent across the PGNN and Gradient Boosting, validating the feature engineering independently of model class. The convergence on $\tau_{QMF}$, β, and γ as top predictors provides actionable guidance: epidemic threshold prediction benefits most from combining the spectral structural baseline with epidemiological parameters, rather than from raw spectral features alone.

## 6 Discussion

Our comparative study yields four overarching insights.

**On predictive accuracy and model selection.** Tree-based ensemble methods (GB: $R^{2}=0.869\pm 0.068$; RF: $R^{2}=0.859\pm 0.084$) substantially outperform the PGNN ($R^{2}=-9.138\pm 17.553$) in the low-data regime. As shown in Section 5.2, this reflects the parameter-to-sample ratio disadvantage and not an inherent limitation of physics-guided modelling.

**On the role of physical constraints as structured regularisers.** The ablation study reveals an ambivalent role: ${ℒ}_{bound}$ is a genuine regulariser (+25.0% RMSE when removed), while ${ℒ}_{stab}$ over-regularises (−28.4% RMSE when removed) because the QMF lower bound is violated by $\tau^{*}$ on ≈18% of networks due to fifinite-size effects. Future work should derive data-adaptive bounds calibrated to the stochastic simulation protocol.

**On the KSEL calibration contribution.** The gradient-based calibration reveals that k^*^ ranges from 0.627 (WS) to 1.322 (REG), departing substantially from the universal constant *k* = 0.3 of prior work. This topology-dependence has direct implications for practitioners applying KSEL to real contact networks.

**On the conditions under which PGNN is preferable.** Three scenarios favour the PGNN over ensemble methods. *First*, out-of-distribution generalisation: physical constraints prevent predictions that violate known epidemiological laws on unseen topologies. *Second*, large-data regimes: neural network and ensemble learning curves are expected to cross at $n\sim 10^{3}-10^{4}$ examples [7,8]; at this scale the PGNN should outperform GB while retaining its interpretability advantages. *Third*, knowledge integration: new theoretical results can be incorporated directly into the PGNN loss function—a modular extensibility that tree-based methods cannot offer.

### 6.1 Why physics-guided models remain relevant

Although tree-based models outperform the proposed PGNN in our experiments, this does not invalidate the relevance of hybrid approaches. Physics-guided models offer three key advantages that purely data-driven methods cannot provide.

**First, structural inductive bias.** The physical constraints ${ℒ}_{stab}$, ${ℒ}_{mono}$, and ${ℒ}_{bound}$ encode known properties of epidemic dynamics derived from the QMF approximation and SIS compartmental theory. In out-of-distribution settings—network topologies not represented in the training data—these constraints prevent the model from producing predictions that violate fundamental epidemiological laws, a guarantee that Gradient Boosting, as a purely data-driven method, cannot provide.

**Second, principled knowledge integration.** When new theoretical results become available (e.g., tighter spectral bounds, heterogeneous SIS corrections, age-structured contact patterns), they can be incorporated directly into the PGNN loss function. This modular extensibility is architecturally impossible for tree-based methods, making the PGNN a natural framework for progressive model refinement as domain knowledge grows.

**Third, interpretability.** Through SHAP attribution, the PGNN provides feature-level insight into how structural and epidemiological factors jointly influence $\tau^{*}$. The convergence of PGNN and Gradient Boosting on the same top predictors ($\tau_{QMF}$, γ, β) validates this interpretability independently of model class.

Therefore, rather than competing directly with classical models in terms of raw predictive performance, physics-guided neural networks should be viewed as complementary tools, particularly suited for scientifically grounded modelling in settings where theoretical consistency and extrapolation safety matter as much as in-sample accuracy.

**Limitations.** Several limitations merit acknowledgment. First, the ground truth $\tau^{*}$ is estimated via stochastic simulation, which introduces Monte Carlo noise; the susceptibility-peak estimator used here is robust for the network families considered (median $\sigma_{\tau^{*}}=0.0072$) but may be sensitive in strongly heterogeneous or periodic regimes; future work could use SIS quasi-stationary distribution methods or multi-criteria estimators (persistence time, final epidemic size, branching factor) for more precise labels. Second, the training set consists of synthetic networks; the model has not been validated on empirical contact networks (workplace, school, hospital settings); such validation is needed before claims of direct applicability can be made, and is identified as a priority for future work. Third, the current model addresses a homogeneous SIS process; extension to SEIR dynamics, age-structured populations, or multiplex networks requires augmenting the feature vector and deriving new physical constraints. Finally, the model assumes β and γ are known, whereas in practice they must be inferred from surveillance data via Bayesian inverse methods.

## 7 Conclusion

We presented a comparative hybrid AI-mathematical framework for epidemic threshold prediction in contact networks. Evaluated on 775 synthetic networks via 5-fold cross-validation repeated over 5 seeds, Gradient Boosting achieves the best predictive accuracy ($R^{2}=0.869\pm 0.068$), while the PGNN ($R^{2}=-9.138\pm 17.553$) exhibits high instability consistent with overfitting in the low-data regime.

Three messages emerge. First, physics-guided constraints are not uniformly beneficial: ${ℒ}_{bound}$ is a genuine regulariser while ${ℒ}_{stab}$ over-regularises, demonstrating that constraint design must account for finite-sample behaviour. Second, gradient-based KSEL calibration reveals topology-dependent optimal values ($k^{*}\in [0.627,1.322]$), substantially departing from the universal constant of prior work. Third, the PGNN offers advantages that tree-based methods cannot provide: out-of-distribution physical consistency, SHAP interpretability, and modular integration of new theoretical knowledge—advantages that become decisive as dataset size grows beyond the current regime.

Future directions include: (i) scaling to n≥2,000 networks to test whether the PGNN closes the performance gap with ensemble methods; (ii) replacing the QMF lower-bound constraint with a tighter, data-adaptive bound; (iii) extending to SEIR dynamics and multiplex networks; and (iv) Bayesian parameter inference for end-to-end prediction from surveillance data.

**Take-home message.** Physics-guided neural models should not be viewed as universally superior predictors, but rather as structured learning frameworks that incorporate domain constraints. In small to moderate datasets, simpler models may achieve higher accuracy, while physics-guided approaches provide interpretability and theoretical consistency. This distinction is essential for the correct use of hybrid models in network epidemiology.

## Supporting information

S1 File Python source code for the hybrid AI-mathematical model. Complete Python implementation (hybrid_model_complete.pdf) of the Physics-Guided Neural Network (PGNN) framework presented in this manuscript, including data generation, model training, cross-validation, ablation study, SHAP interpretability analysis, and automatic KSEL coefficient calibration. The code is also available at: https://doi.org/10.5281/zenodo.19411519 (CC BY 4.0).
(PDF)
