# Supplementary material for: A Hybrid AI-Mathematical approach for epidemic threshold prediction in metapopulation networks: Integrating physics-guided neural networks with spectral graph theory
Source: PLoS One. 2026 Jun 18;21(6):e0344827. doi: 10.1371/journal.pone.0344827 (PMC13278587; doi:10.1371/journal.pone.0344827)
Supplement: S1 File — Complete Python implementation (hybrid_model_complete.pdf) of the Physics-Guided Neural Network (PGNN) framework presented in this manuscript, including data generation, model training, cross-validation, ablation study, SHAP interpretability analysis, and automatic KSEL coefficient calibration. The code is also available at: https://doi.org/10.5281/zenodo.19411519 (CC BY 4.0). (PDF) [file pone.0344827.s001.pdf]

Supporting Information S1 -- Python Source Code

File: hybrid_model_complete.py

Manuscript: PONE-D-26-09719R2 -- Kouokam (2026), PLOS ONE

Zenodo: https://doi.org/10.5281/zenodo.19411519 (CC BY 4.0)

```
#!/usr/bin/env python3
"""
============================================================================
Hybrid AI-Mathematical Approach for Epidemic Threshold Prediction
============================================================================
Author  : Etienne E. KOUOKAM
Affil.  : University of Yaoundé I, Cameroon / IRD-UMMISCO, Sorbonne U., France
Date    : February 2026

Description
-----------
Complete implementation of the hybrid PGNN model for epidemic threshold ?
prediction in metapopulation networks. The pipeline covers:
  1. Synthetic network dataset generation (Erd?s-Rényi, Barabási-Albert,
     Watts-Strogatz, regular)
  2. Ground-truth ? estimation via stochastic SIS simulation
  3. Spectral + topological feature extraction
  4. Physics-Guided Neural Network (PGNN) training with hybrid loss
  5. Automatic gradient-based KSEL coefficient calibration
  6. SHAP interpretability analysis
  7. Performance evaluation and comparison

Requirements
------------
  numpy, scipy, networkx, matplotlib, torch (>=1.9), sklearn
  Optional: shap  (pip install shap)

Usage
-----
  python hybrid_model_complete.py            # full pipeline
  python hybrid_model_complete.py --demo     # fast demo on 50 networks
============================================================================
"""

import argparse
import numpy as np
import warnings
warnings.filterwarnings("ignore")

# ?? Standard library ??????????????????????????????????????????????????????????
import os
import time
import json
from pathlib import Path

# ?? Scientific stack ??????????????????????????????????????????????????????????
import numpy as np
import scipy.sparse as sp
from scipy.sparse.linalg import eigsh

# ?? Network library ???????????????????????????????????????????????????????????
import networkx as nx

# ?? Visualisation ?????????????????????????????????????????????????????????????
import matplotlib
matplotlib.use("Agg")
import matplotlib.pyplot as plt
import matplotlib.gridspec as gridspec

# ?? Sklearn ???????????????????????????????????????????????????????????????????
from sklearn.model_selection import KFold
```

```
from sklearn.metrics import mean_squared_error, mean_absolute_error, r2_score
from sklearn.preprocessing import StandardScaler
from sklearn.linear_model import Ridge

# ?? PyTorch ??????????????????????????????????????????????????????????????
import torch
import torch.nn as nn
import torch.optim as optim
from torch.utils.data import DataLoader, TensorDataset

# ?? Optional SHAP ????????????????????????????????????????????????????????????
try:
    import shap
    HAS_SHAP = True
except ImportError:
    HAS_SHAP = False
    print("[WARNING] shap not installed - using manual feature importance proxy.")

# ??????????????????????????????????????????????????????????????????????????????
# CONFIGURATION
# ??????????????????????????????????????????????????????????????????????????????
SEED = 42
np.random.seed(SEED)
torch.manual_seed(SEED)

DEVICE = torch.device("cuda" if torch.cuda.is_available() else "cpu")

# Output directories
OUT_DIR = Path("output")
FIG_DIR = OUT_DIR / "figures"
DATA_DIR = OUT_DIR / "data"
for d in [OUT_DIR, FIG_DIR, DATA_DIR]:
    d.mkdir(parents=True, exist_ok=True)


# ??????????????????????????????????????????????????????????????????????????????
# SECTION 1 - SPECTRAL FEATURE EXTRACTION
# ??????????????????????????????????????????????????????????????????????????????

def spectral_features(G: nx.Graph) -> dict:
    """
    Compute the 9 structural features of network G:
      lambda_max     - spectral radius of adjacency matrix
      lambda2        - algebraic connectivity (2nd smallest Laplacian eigenvalue)
      laplacian_energy - Laplacian energy LE(G) = ?|?_i - 2m/n|
      n, m           - number of nodes and edges
      mean_degree    - <k>
      mean_degree2   - <k²>
      clustering     - mean clustering coefficient
      diameter       - network diameter (may be ? for disconnected)
    """
    n = G.number_of_nodes()
    m = G.number_of_edges()
    if n == 0 or m == 0:
        return {k: 0.0 for k in ["lambda_max", "lambda2", "laplacian_energy",
                                  "n", "m", "mean_degree", "mean_degree2",
                                  "clustering", "diameter"]}

    A = nx.to_scipy_sparse_array(G, format="csr", dtype=float)
    L = sp.diags([d for _, d in G.degree()]) - A

    # Spectral radius of A
    try:
        lambda_max = float(eigsh(A, k=1, which="LM", return_eigenvectors=False)[0])
    except Exception:
        lambda_max = max(nx.adjacency_spectrum(G).real)

    # Laplacian eigenvalues
```

```
    try:
        k_eig = min(n - 1, 6)
        mu_all = eigsh(L, k=k_eig, which="SM", return_eigenvectors=False)
        mu_all = np.sort(mu_all.real)
        lambda2 = float(mu_all[1]) if len(mu_all) > 1 else 0.0
    except Exception:
        L_dense = L.toarray()
        mu_all = np.linalg.eigvalsh(L_dense)
        lambda2 = float(sorted(mu_all)[1]) if n > 1 else 0.0
        mu_all = mu_all

    # Full Laplacian energy (use dense for small networks)
    if n <= 500:
        L_dense = L.toarray() if sp.issparse(L) else L
        mu_full = np.linalg.eigvalsh(L_dense)
    else:
        mu_full = mu_all  # approximate
    mean_mu = 2 * m / n
    laplacian_energy = float(np.sum(np.abs(mu_full - mean_mu)))

    degrees = [d for _, d in G.degree()]
    mean_k  = np.mean(degrees)
    mean_k2 = np.mean([d**2 for d in degrees])

    try:
        clustering = nx.average_clustering(G)
    except Exception:
        clustering = 0.0

    if nx.is_connected(G):
        try:
            diameter = float(nx.diameter(G))
        except Exception:
            diameter = float(n)
    else:
        try:
            diameter = float(max(
                nx.diameter(G.subgraph(c)) for c in nx.connected_components(G)
            ))
        except Exception:
            diameter = float(n)

    return {
        "lambda_max": lambda_max,
        "lambda2": lambda2,
        "laplacian_energy": laplacian_energy,
        "n": float(n),
        "m": float(m),
        "mean_degree": mean_k,
        "mean_degree2": mean_k2,
        "clustering": clustering,
        "diameter": diameter,
    }


def tau_qmf(features: dict) -> float:
    """QMF estimator: ? = 1/?_max."""
    return 1.0 / max(features["lambda_max"], 1e-6)


def tau_ksel(features: dict, k: float = 0.3) -> float:
    """KSEL estimator: ? = k·n / LE(G) · exp(?1/?_max)."""
    lam = max(features["lambda_max"], 1e-6)
    le  = max(features["laplacian_energy"], 1e-6)
    return k * features["n"] / le * np.exp(-1.0 / lam)


# ??????????????????????????????????????????????????????????????????????????????
```

```
# SECTION 2 - STOCHASTIC SIS SIMULATION (ground truth ?)
# ???????????????????????????????????????????????????????????????????????????????

def sis_epidemic_probability(G: nx.Graph,
                              beta: float,
                              gamma: float,
                              tau_test: float,
                              n_sims: int = 500,
                              t_max: int = 200) -> float:
    """
    Estimate P(epidemic | ?_test) via Monte Carlo SIS simulation.
    Returns fraction of runs where final infected fraction > 0.01.
    """
    nodes = list(G.nodes())
    n = len(nodes)
    adj = {v: list(G.neighbors(v)) for v in nodes}
    beta_test = tau_test * gamma
    epidemic_count = 0

    for _ in range(n_sims):
        state = np.zeros(n, dtype=int)
        state[np.random.randint(n)] = 1  # seed one infected

        for _ in range(t_max):
            new_state = state.copy()
            infected = np.where(state == 1)[0]
            if len(infected) == 0:
                break
            for i in infected:
                # Recovery
                if np.random.rand() < gamma:
                    new_state[i] = 0
                # Transmission
                for j in adj[nodes[i]]:
                    j_idx = nodes.index(j)
                    if state[j_idx] == 0 and np.random.rand() < beta_test:
                        new_state[j_idx] = 1
            state = new_state

        final_infected = np.sum(state) / n
        if final_infected > 0.01:
            epidemic_count += 1

    return epidemic_count / n_sims


def find_epidemic_threshold(G: nx.Graph,
                             beta: float,
                             gamma: float,
                             tau_min: float = None,
                             tau_max: float = None,
                             n_sims: int = 300,
                             tol: float = 0.02) -> float:
    """
    Binary search for ?* such that P(epidemic | ?*) ? 0.5.
    Faster approximation: use QMF as prior and search in [?_qmf/2, ?_qmf*3].
    """
    feats = spectral_features(G)
    tau_q = tau_qmf(feats)
    if tau_min is None:
        tau_min = max(tau_q * 0.3, 1e-4)
    if tau_max is None:
        tau_max = min(tau_q * 4.0, beta / max(gamma, 1e-6))

    # Bisection
    for _ in range(12):
        tau_mid = (tau_min + tau_max) / 2.0
        p = sis_epidemic_probability(G, beta, gamma, tau_mid, n_sims=n_sims)
```

```
        if p < 0.5:
            tau_min = tau_mid
        else:
            tau_max = tau_mid
        if tau_max - tau_min < tol:
            break

    return (tau_min + tau_max) / 2.0


# ?????????????????????????????????????????????????????????????????????????????
# SECTION 3 - DATASET GENERATION
# ?????????????????????????????????????????????????????????????????????????????

def generate_network(family: str, n: int, **params) -> nx.Graph:
    """Generate a network of a given family."""
    if family == "erdos_renyi":
        p = params.get("p", 0.1)
        G = nx.erdos_renyi_graph(n, p, seed=np.random.randint(1e6))
    elif family == "barabasi_albert":
        m0 = params.get("m0", 3)
        G = nx.barabasi_albert_graph(n, m0, seed=np.random.randint(1e6))
    elif family == "watts_strogatz":
        k = params.get("k", 6)
        p_r = params.get("p_r", 0.1)
        G = nx.watts_strogatz_graph(n, k, p_r, seed=np.random.randint(1e6))
    elif family == "regular":
        d = params.get("d", 4)
        if d >= n:
            d = max(2, n // 4)
        if (n * d) % 2 != 0:
            d = max(2, d - 1)
        try:
            G = nx.random_regular_graph(d, n, seed=np.random.randint(1e6))
        except Exception:
            G = nx.erdos_renyi_graph(n, 0.1, seed=np.random.randint(1e6))
    else:
        raise ValueError(f"Unknown network family: {family}")

    # Ensure connectivity for simulation
    if not nx.is_connected(G):
        components = list(nx.connected_components(G))
        for i in range(len(components) - 1):
            u = np.random.choice(list(components[i]))
            v = np.random.choice(list(components[i+1]))
            G.add_edge(u, v)

    return G



def build_dataset(n_networks: int = 1200, quick_sim: bool = False) -> list:
    """
    Build the full dataset of (features, tau_true, tau_qmf, tau_ksel) tuples.

    Parameters
    ----------
    n_networks : total number of networks (divided equally among 4 families)
    quick_sim  : if True, use QMF as proxy for tau_true (fast, for demo)
    """
    per_family = n_networks // 4
    families = ["erdos_renyi", "barabasi_albert", "watts_strogatz", "regular"]
    dataset = []

    for family in families:
        print(f"  Generating {per_family} {family} networks...")
        for i in range(per_family):
            n = np.random.randint(50, 301)
            # Sample family-specific parameters
```

```
            if family == "erdos_renyi":
                p = np.random.uniform(0.01, 0.30)
                G = generate_network(family, n, p=p)
            elif family == "barabasi_albert":
                m0 = np.random.randint(2, 8)
                G = generate_network(family, n, m0=m0)
            elif family == "watts_strogatz":
                k = np.random.randint(4, 12)
                p_r = np.random.uniform(0.01, 0.5)
                G = generate_network(family, n, k=k, p_r=p_r)
            else:  # regular
                d = np.random.randint(2, 8)
                G = generate_network(family, n, d=d)

            feats = spectral_features(G)

            beta  = np.random.uniform(0.10, 1.00)
            gamma = np.random.uniform(0.05, 0.50)

            if quick_sim:
                # Fast proxy: add noise to QMF
                tau_true = tau_qmf(feats) * np.random.uniform(0.7, 1.3)
            else:
                tau_true = find_epidemic_threshold(G, beta, gamma, n_sims=200)

            record = {
                "family": family,
                "features": feats,
                "beta": beta,
                "gamma": gamma,
                "ratio_bg": beta / gamma,
                "tau_true": tau_true,
                "tau_qmf_val": tau_qmf(feats),
                "tau_ksel_val": tau_ksel(feats, k=0.3),
            }
            dataset.append(record)

            if (i + 1) % 50 == 0:
                print(f"    [{family}] {i+1}/{per_family} done")

    return dataset


def dataset_to_arrays(dataset: list):
    """Convert list of records to (X, y, tau_qmf, tau_ksel, families)."""
    feature_keys = ["lambda_max", "lambda2", "laplacian_energy",
                    "n", "m", "mean_degree", "mean_degree2",
                    "clustering", "diameter"]

    X, y, tau_qmf_arr, tau_ksel_arr, families = [], [], [], [], []
    for rec in dataset:
        row = [rec["features"][k] for k in feature_keys]
        row += [rec["beta"], rec["gamma"], rec["ratio_bg"], rec["tau_qmf_val"]]
        X.append(row)
        y.append(rec["tau_true"])
        tau_qmf_arr.append(rec["tau_qmf_val"])
        tau_ksel_arr.append(rec["tau_ksel_val"])
        families.append(rec["family"])

    return (np.array(X, dtype=np.float32),
            np.array(y, dtype=np.float32),
            np.array(tau_qmf_arr, dtype=np.float32),
            np.array(tau_ksel_arr, dtype=np.float32),
            np.array(families))


FEATURE_NAMES = [
    r"$\lambda_{\max}$",
```

```
    r"$\lambda_2$",
    r"$LE(G)$",
    r"$n$",
    r"$m$",
    r"$\langle k\rangle$",
    r"$\langle k^2\rangle$",
    r"$\bar{C}$",
    r"$\mathrm{diam}$",
    r"$\beta$",
    r"$\gamma$",
    r"$\beta/\gamma$",
    r"$\tau_{\mathrm{QMF}}$",
]


# ??????????????????????????????????????????????????????????????????????????????
# SECTION 4 - PHYSICS-INFORMED NEURAL NETWORK
# ??????????????????????????????????????????????????????????????????????????????

class PGNN(nn.Module):
    """
    Physics-Guided Neural Network for epidemic threshold prediction.

    Architecture: BatchNorm ? [128 ? 64 ? 32] (ReLU + Dropout + BN) ? Softplus

    The physical constraints are enforced in the training loss, not in the
    architecture itself, to preserve differentiability.
    """

    def __init__(self, input_dim: int = 13, dropout: float = 0.2):
        super().__init__()
        self.bn_input = nn.BatchNorm1d(input_dim)

        self.layers = nn.Sequential(
            nn.Linear(input_dim, 128),
            nn.BatchNorm1d(128),
            nn.ReLU(),
            nn.Dropout(dropout),

            nn.Linear(128, 64),
            nn.BatchNorm1d(64),
            nn.ReLU(),
            nn.Dropout(dropout),

            nn.Linear(64, 32),
            nn.BatchNorm1d(32),
            nn.ReLU(),
            nn.Dropout(dropout),

            nn.Linear(32, 1),
            nn.Softplus(),      # ensures ?? > 0
        )

    def forward(self, x: torch.Tensor) -> torch.Tensor:
        x = self.bn_input(x)
        return self.layers(x).squeeze(-1)


def physics_loss(tau_pred: torch.Tensor,
                 X: torch.Tensor,
                 lambda_phys: float = 0.1) -> torch.Tensor:
    """
    Three physical constraints:
      1. Stability:    ?? ? ?_QMF = 1/?_max  (soft, via ReLU penalty)
      2. Monotonicity: not enforced here (handled via weight regularisation)
      3. Boundedness:  ?? ? ?/?              (soft penalty)

    Parameters
```

```
    ----------
    tau_pred  : model predictions (batch_size,)
    X         : input features (batch_size, 13)
                column 0  = ?_max
                column 11 = ?/?
    """
    eps = 1e-6

    lambda_max = X[:, 0].clamp(min=eps)
    tau_qmf_lower = 1.0 / lambda_max
    ratio_bg = X[:, 11].clamp(min=eps)

    # Constraint 1: ?? ? ?_QMF  ?  penalty if ?? < ?_QMF
    c1 = torch.relu(tau_qmf_lower - tau_pred).mean()

    # Constraint 3: ?? ? ?/?  ?  penalty if ?? > ?/?
    c3 = torch.relu(tau_pred - ratio_bg).mean()

    return lambda_phys * (c1 + c3)


def train_pinn(X_train: np.ndarray,
               y_train: np.ndarray,
               X_val: np.ndarray,
               y_val: np.ndarray,
               lambda_phys: float = 0.1,
               lr: float = 1e-3,
               n_epochs: int = 200,
               batch_size: int = 64,
               patience: int = 20) -> tuple:
    """
    Train PGNN with hybrid loss. Returns (model, scaler, train_history).
    """
    # Feature scaling
    scaler = StandardScaler()
    X_tr = scaler.fit_transform(X_train).astype(np.float32)
    X_va = scaler.transform(X_val).astype(np.float32)

    # Tensors
    Xtr_t = torch.tensor(X_tr).to(DEVICE)
    ytr_t = torch.tensor(y_train.astype(np.float32)).to(DEVICE)
    Xva_t = torch.tensor(X_va).to(DEVICE)
    yva_t = torch.tensor(y_val.astype(np.float32)).to(DEVICE)

    loader = DataLoader(TensorDataset(Xtr_t, ytr_t),
                        batch_size=batch_size, shuffle=True)

    model = PGNN(input_dim=X_train.shape[1]).to(DEVICE)
    optimiser = optim.Adam(model.parameters(), lr=lr)
    scheduler = optim.lr_scheduler.ExponentialLR(optimiser, gamma=0.95)

    best_val_loss = np.inf
    best_state = None
    no_improve = 0
    history = {"train_loss": [], "val_loss": []}

    for epoch in range(n_epochs):
        model.train()
        epoch_loss = 0.0
        for Xb, yb in loader:
            optimiser.zero_grad()
            pred = model(Xb)
            loss = nn.MSELoss()(pred, yb) + physics_loss(pred, Xb, lambda_phys)
            loss.backward()
            optimiser.step()
            epoch_loss += loss.item() * len(Xb)
        epoch_loss /= len(X_train)
```

```
        model.eval()
        with torch.no_grad():
            val_pred = model(Xva_t)
            val_loss = nn.MSELoss()(val_pred, yva_t).item()

        history["train_loss"].append(epoch_loss)
        history["val_loss"].append(val_loss)

        if val_loss < best_val_loss:
            best_val_loss = val_loss
            best_state = {k: v.clone() for k, v in model.state_dict().items()}
            no_improve = 0
        else:
            no_improve += 1
            if no_improve >= patience:
                break

        if (epoch + 1) % 20 == 0:
            print(f"    Epoch {epoch+1:3d} | train={epoch_loss:.5f}"
                  f" | val={val_loss:.5f}")

        if (epoch + 1) % 20 == 0:
            scheduler.step()

    if best_state is not None:
        model.load_state_dict(best_state)

    return model, scaler, history


def predict(model: nn.Module,
            scaler: StandardScaler,
            X: np.ndarray) -> np.ndarray:
    """Run inference on a numpy array, return numpy predictions."""
    model.eval()
    X_sc = scaler.transform(X).astype(np.float32)
    with torch.no_grad():
        pred = model(torch.tensor(X_sc).to(DEVICE)).cpu().numpy()
    return pred


# ??????????????????????????????????????????????????????????????????????????????
# SECTION 5 - KSEL AUTOMATIC CALIBRATION
# ??????????????????????????????????????????????????????????????????????????????

def calibrate_ksel(X_train: np.ndarray,
                   y_train: np.ndarray,
                   lr: float = 0.01,
                   n_steps: int = 200) -> float:
    """
    Gradient-based calibration of the KSEL coefficient k.

    The KSEL formula: ?_KSEL(k) = k * n / LE(G) * exp(-1/?_max)
    We differentiate through it and minimise MSE over training set.

    Column indices in X:
      0  = ?_max
      2  = LE(G)
      3  = n
    """
    lam_max = torch.tensor(X_train[:, 0], dtype=torch.float32)
    le_g    = torch.tensor(X_train[:, 2], dtype=torch.float32).clamp(min=1e-6)
    n_vec   = torch.tensor(X_train[:, 3], dtype=torch.float32)
    y_t     = torch.tensor(y_train, dtype=torch.float32)

    k = torch.tensor([0.3], dtype=torch.float32, requires_grad=True)
    opt = optim.Adam([k], lr=lr)
```

```
    for step in range(n_steps):
        opt.zero_grad()
        tau_ksel = k * n_vec / le_g * torch.exp(-1.0 / lam_max.clamp(min=1e-6))
        loss = nn.MSELoss()(tau_ksel, y_t)
        loss.backward()
        opt.step()
        k.data.clamp_(min=0.05, max=2.0)  # keep k in plausible range

    return float(k.item())


# ?????????????????????????????????????????????????????????????????????????????
# SECTION 6 - EVALUATION METRICS
# ?????????????????????????????????????????????????????????????????????????????

def compute_metrics(y_true: np.ndarray, y_pred: np.ndarray) -> dict:
    """Compute RMSE, MAE, R²."""
    return {
        "RMSE": float(np.sqrt(mean_squared_error(y_true, y_pred))),
        "MAE":  float(mean_absolute_error(y_true, y_pred)),
        "R2":   float(r2_score(y_true, y_pred)),
    }


def evaluate_all_methods(X_test: np.ndarray,
                          y_test: np.ndarray,
                          tau_qmf_test: np.ndarray,
                          tau_ksel_test: np.ndarray,
                          tau_ksel_calib: np.ndarray,
                          model_pinn: nn.Module,
                          scaler_pinn: StandardScaler,
                          model_mlp: nn.Module = None,
                          scaler_mlp: StandardScaler = None) -> dict:
    """Evaluate all methods on the test set."""
    results = {}
    results["QMF"]  = compute_metrics(y_test, tau_qmf_test)
    results["KSEL (k=0.3)"] = compute_metrics(y_test, tau_ksel_test)
    results["KSEL (k*)"] = compute_metrics(y_test, tau_ksel_calib)

    # Linear regression baseline (already fitted before call)
    pinn_pred = predict(model_pinn, scaler_pinn, X_test)
    results["PGNN"] = compute_metrics(y_test, pinn_pred)

    if model_mlp is not None:
        mlp_pred = predict(model_mlp, scaler_mlp, X_test)
        results["MLP (no physics)"] = compute_metrics(y_test, mlp_pred)

    return results


# ?????????????????????????????????????????????????????????????????????????????
# SECTION 7 - SHAP / FEATURE IMPORTANCE
# ?????????????????????????????????????????????????????????????????????????????

def compute_shap_importance(model: nn.Module,
                             scaler: StandardScaler,
                             X_background: np.ndarray,
                             X_explain: np.ndarray) -> np.ndarray:
    """
    Compute SHAP values using DeepExplainer (PyTorch).
    Falls back to gradient-based importance if shap is unavailable.
    """
    if HAS_SHAP:
        model.eval()
        X_bg = torch.tensor(scaler.transform(X_background).astype(np.float32)).to(DEVICE)
        X_ex = torch.tensor(scaler.transform(X_explain).astype(np.float32)).to(DEVICE)
        explainer = shap.DeepExplainer(model, X_bg)
        shap_values = np.array(explainer.shap_values(X_ex))
```

```
        return np.abs(shap_values).mean(axis=0)
    else:
        # Proxy: gradient × input sensitivity
        model.eval()
        X_ex = torch.tensor(scaler.transform(X_explain).astype(np.float32),
                            requires_grad=True).to(DEVICE)
        out = model(X_ex).sum()
        out.backward()
        importance = X_ex.grad.abs().mean(dim=0).cpu().numpy()
        # Normalise to sum to 1
        importance = importance / importance.sum()
        return importance


# ?????????????????????????????????????????????????????????????????????????????
# SECTION 8 - PLOTTING
# ?????????????????????????????????????????????????????????????????????????????

def plot_training_history(history: dict, save_path: Path):
    fig, ax = plt.subplots(figsize=(8, 4))
    ax.plot(history["train_loss"], label="Train loss", color="steelblue")
    ax.plot(history["val_loss"],   label="Val loss",   color="tomato")
    ax.set_xlabel("Epoch"); ax.set_ylabel("MSE Loss")
    ax.set_title("PGNN Training History")
    ax.legend(); ax.grid(True, alpha=0.3)
    plt.tight_layout()
    plt.savefig(save_path, dpi=150)
    plt.close()


def plot_predictions(y_true: np.ndarray,
                     y_qmf: np.ndarray,
                     y_ksel: np.ndarray,
                     y_pinn: np.ndarray,
                     save_path: Path):
    fig, axes = plt.subplots(1, 3, figsize=(14, 5))
    fig.suptitle("Epidemic Threshold Predictions vs True Values",
                 fontsize=14, fontweight="bold")
    mn, mx = 0, max(y_true.max(), 0.8)

    for ax, (pred, name, color, r2) in zip(axes, [
        (y_qmf,  "QMF",       "royalblue",  r2_score(y_true, y_qmf)),
        (y_ksel, "KSEL",      "darkorange", r2_score(y_true, y_ksel)),
        (y_pinn, "PGNN (ours)","crimson",   r2_score(y_true, y_pinn)),
    ]):
        rmse = np.sqrt(mean_squared_error(y_true, pred))
        ax.scatter(y_true, pred, c=color, alpha=0.5, s=18, edgecolors="white", lw=0.3)
        ax.plot([mn, mx], [mn, mx], "k--", lw=1.5)
        ax.set_xlabel(r"True $\tau$"); ax.set_ylabel(r"Predicted $\hat{\tau}$")
        ax.set_title(f"{name}\nRMSE={rmse:.4f}, R²={r2:.3f}")
        ax.set_xlim(mn, mx); ax.set_ylim(mn, mx)
        ax.set_aspect("equal"); ax.grid(True, alpha=0.3)
    plt.tight_layout()
    plt.savefig(save_path, dpi=150)
    plt.close()


def plot_shap(importance: np.ndarray,
              feature_names: list,
              save_path: Path):
    idx_sorted = np.argsort(importance)
    fig, ax = plt.subplots(figsize=(9, 5))
    colors = plt.cm.viridis(np.linspace(0.2, 0.9, len(idx_sorted)))
    ax.barh([feature_names[i] for i in idx_sorted],
            importance[idx_sorted],
            color=colors[::-1], edgecolor="white")
    ax.set_xlabel("Mean |SHAP value|", fontsize=12)
    ax.set_title("Feature Importance - SHAP Analysis", fontsize=13, fontweight="bold")
```

```
    ax.grid(axis="x", alpha=0.3)
    plt.tight_layout()
    plt.savefig(save_path, dpi=150)
    plt.close()


def print_results_table(results: dict):
    print("\n" + "=" * 65)
    print(f"{'Method':<25} {'RMSE':>8} {'MAE':>8} {'R²':>8}")
    print("-" * 65)
    for name, m in results.items():
        print(f"{name:<25} {m['RMSE']:>8.4f} {m['MAE']:>8.4f} {m['R2']:>8.3f}")
    print("=" * 65)


# ?????????????????????????????????????????????????????????????????????????????
# SECTION 9 - MAIN PIPELINE
# ?????????????????????????????????????????????????????????????????????????????

def main(demo_mode: bool = False):
    n_networks = 200 if demo_mode else 1200
    quick_sim  = demo_mode  # use QMF proxy if in demo mode

    print("=" * 65)
    print("  Hybrid AI-Math Epidemic Threshold Prediction - PGNN")
    print(f"  Mode: {'DEMO (fast)' if demo_mode else 'FULL'}")
    print(f"  Networks: {n_networks} | Device: {DEVICE}")
    print("=" * 65)

    # ?? 1. Dataset generation ?????????????????????????????????????????????
    print("\n[1/6] Generating synthetic network dataset...")
    t0 = time.time()
    dataset = build_dataset(n_networks=n_networks, quick_sim=quick_sim)
    print(f"  Done in {time.time()-t0:.1f}s. Total records: {len(dataset)}")

    X, y, tau_qmf_arr, tau_ksel_arr, families = dataset_to_arrays(dataset)
    print(f"  Feature matrix: {X.shape}, y shape: {y.shape}")

    # Save raw data
    np.savez(DATA_DIR / "dataset.npz",
             X=X, y=y,
             tau_qmf=tau_qmf_arr,
             tau_ksel=tau_ksel_arr,
             families=families)
    print(f"  Dataset saved to {DATA_DIR / 'dataset.npz'}")

    # ?? 2. Train/val/test split ???????????????????????????????????????????
    n_total = len(X)
    idx = np.random.permutation(n_total)
    n_train = int(0.70 * n_total)
    n_val   = int(0.15 * n_total)

    tr_idx   = idx[:n_train]
    val_idx  = idx[n_train:n_train+n_val]
    test_idx = idx[n_train+n_val:]

    X_train, y_train = X[tr_idx],   y[tr_idx]
    X_val,   y_val   = X[val_idx],  y[val_idx]
    X_test,  y_test  = X[test_idx], y[test_idx]

    tq_test  = tau_qmf_arr[test_idx]
    tk_test  = tau_ksel_arr[test_idx]

    print(f"\n  Split ? train={len(X_train)}, val={len(X_val)}, test={len(X_test)}")

    # ?? 3. KSEL automatic calibration ????????????????????????????????????
    print("\n[2/6] Calibrating KSEL coefficient k via gradient descent...")
    k_star = calibrate_ksel(X_train, y_train)
```

```
    print(f"  Optimal k* = {k_star:.4f}  (default = 0.3000)")

    # Recompute KSEL with k*
    lam = np.maximum(X_test[:, 0], 1e-6)
    le  = np.maximum(X_test[:, 2], 1e-6)
    n_v = X_test[:, 3]
    tk_test_calib = k_star * n_v / le * np.exp(-1.0 / lam)

    # ?? 4. Train PGNN ????????????????????????????????????????????????????????
    n_epochs = 50 if demo_mode else 200
    print(f"\n[3/6] Training PGNN (max {n_epochs} epochs, ?_phys=0.1)...")
    model_pinn, scaler_pinn, history_pinn = train_pinn(
        X_train, y_train, X_val, y_val,
        lambda_phys=0.1, lr=1e-3, n_epochs=n_epochs, patience=20
    )
    plot_training_history(history_pinn, FIG_DIR / "training_history_pgnn.png")
    print(f"  PGNN training complete.")

    # ?? 5. Train standard MLP (no physics) ???????????????????????????????
    print("\n[4/6] Training standard MLP (no physics)...")
    model_mlp, scaler_mlp, _ = train_pinn(
        X_train, y_train, X_val, y_val,
        lambda_phys=0.0, lr=1e-3, n_epochs=n_epochs, patience=20
    )

    # ?? 6. Evaluate ??????????????????????????????????????????????????????????
    print("\n[5/6] Evaluating all methods on test set...")
    results = evaluate_all_methods(
        X_test, y_test,
        tau_qmf_test=tq_test,
        tau_ksel_test=tk_test,
        tau_ksel_calib=tk_test_calib,
        model_pinn=model_pinn, scaler_pinn=scaler_pinn,
        model_mlp=model_mlp, scaler_mlp=scaler_mlp,
    )
    print_results_table(results)

    pinn_pred = predict(model_pinn, scaler_pinn, X_test)
    rmse_improvement_qmf  = (results["QMF"]["RMSE"] - results["PGNN"]["RMSE"]) \
                            / results["QMF"]["RMSE"] * 100
    rmse_improvement_ksel = (results["KSEL (k=0.3)"]["RMSE"] - results["PGNN"]["RMSE"]) \
                            / results["KSEL (k=0.3)"]["RMSE"] * 100
    print(f"\n  PGNN RMSE reduction vs QMF:  {rmse_improvement_qmf:.1f}%")
    print(f"  PGNN RMSE reduction vs KSEL: {rmse_improvement_ksel:.1f}%")

    # ?? 7. Plots ?????????????????????????????????????????????????????????????
    print("\n[6/6] Generating figures...")
    plot_predictions(y_test, tq_test, tk_test, pinn_pred,
                     FIG_DIR / "predictions_comparison.png")

    # SHAP
    n_bg = min(100, len(X_train))
    X_bg = X_train[:n_bg]
    importance = compute_shap_importance(model_pinn, scaler_pinn, X_bg, X_test[:50])
    plot_shap(importance, FEATURE_NAMES, FIG_DIR / "shap_importance.png")

    # Save results JSON
    with open(OUT_DIR / "results.json", "w") as f:
        json.dump({k: {kk: round(vv, 6) for kk, vv in v.items()}
                   for k, v in results.items()}, f, indent=2)

    print(f"\n  All figures saved to {FIG_DIR}/")
    print(f"  Results JSON saved to {OUT_DIR}/results.json")
    print("\n  Pipeline complete.")


# ?????????????????????????????????????????????????????????????????????????????
# SECTION 10 - STANDALONE UTILITIES (importable)
```

```
# ??????????????????????????????????????????????????????????????????????????????

def predict_tau(G: nx.Graph,
                beta: float,
                gamma: float,
                model: nn.Module,
                scaler: StandardScaler) -> dict:
    """
    User-facing convenience function: predict ? for a given network and
    epidemiological parameters using the trained PGNN, QMF, and KSEL.

    Parameters
    ----------
    G     : networkx Graph
    beta  : infection rate
    gamma : recovery rate
    model : trained PGNN
    scaler: fitted StandardScaler from training

    Returns
    -------
    dict with keys 'PGNN', 'QMF', 'KSEL'
    """
    feats = spectral_features(G)
    row = [feats["lambda_max"], feats["lambda2"], feats["laplacian_energy"],
           feats["n"], feats["m"], feats["mean_degree"], feats["mean_degree2"],
           feats["clustering"], feats["diameter"],
           beta, gamma, beta/gamma, tau_qmf(feats)]
    X = np.array([row], dtype=np.float32)
    tau_pinn_val = float(predict(model, scaler, X)[0])
    return {
        "PGNN":  tau_pinn_val,
        "QMF":   tau_qmf(feats),
        "KSEL":  tau_ksel(feats, k=0.3),
    }


# ??????????????????????????????????????????????????????????????????????????????
if __name__ == "__main__":
    parser = argparse.ArgumentParser(
        description="Hybrid PGNN model for epidemic threshold prediction")
    parser.add_argument("--demo", action="store_true",
                        help="Run a fast demo on 200 networks using QMF proxy labels")
    args = parser.parse_args()
    main(demo_mode=args.demo)
```
