# Supplementary material for: A Hybrid AI-Mathematical approach for epidemic threshold prediction in metapopulation networks: Integrating physics-guided neural networks with spectral graph theory
Source: PLoS One. 2026 Jun 18;21(6):e0344827. doi: 10.1371/journal.pone.0344827 (PMC13278587; doi:10.1371/journal.pone.0344827)
Supplement: Attachment — Submitted filename: Response_to_Reviewers_R2_Final_PONE_D_26_09719_v2.docx [file pone.0344827.s003.docx]

**Response to Reviewers — Revision R2 (Final Submission)**

Manuscript: PONE-D-26-09719 | PLOS ONE

Title: A Hybrid AI-Mathematical Approach for Epidemic Threshold Prediction in Metapopulation Networks

Author: Etienne Kouokam, Université de Yaoundé I, Cameroon | April 2026

Dear Dr. Cunha Jr and Members of the Editorial Board,

We sincerely thank the Academic Editor and both reviewers for their careful and constructive evaluations. We have revised the manuscript thoroughly and believe that all concerns have been fully addressed.

In this final revision, the manuscript has been strengthened in five key areas:

**(i) Main contribution explicitly repositioned** as a systematic methodological evaluation — not a performance-driven proposal (Introduction, p. 2, lines 180–191);

**(ii) Full statistical robustness protocol** with 5-fold CV × 5 seeds = 25 evaluations per method, mean ± std reported throughout (Section 4.2, p. 7, lines 502–509);

**(iii) New subsection: 'Why Physics-Guided Models Remain Relevant'** with three specific arguments — inductive bias, knowledge integration, interpretability (Section 6.1, p. 12);

**(iv) New subsection 5.2 explaining PGNN underperformance** via parameter-to-sample ratio analysis (252 vs 88 for GB), cross-validation instability (8.7× higher std), and physical constraint misspecification with finite-size correction (p. 10);

**(v) Code and data publicly deposited on Zenodo** (DOI: https://doi.org/10.5281/zenodo.19411519 — to be finalised upon upload, pre-publication access available on request).

We provide below a complete, point-by-point response. Each response includes the exact section, page, and line numbers of the corresponding changes in the revised manuscript.

Sincerely,

**Etienne Kouokam**

etienne.kouokam@facsciences-uy1.cm

Department of Computer Science, Université de Yaoundé I, Cameroon

IRD-UMMISCO, Sorbonne Université, France

**Response to Reviewer 1**

**Comment 1.1 — Incremental novelty; synthetic-only evaluation**

**Reviewer comment:**

*The novelty is somewhat incremental, and the evaluation relies entirely on synthetic data with simulation-based ground truth, limiting evidence of real-world applicability.*

**Response:**

We appreciate this important observation and have substantially revised the positioning of the manuscript accordingly. We fully agree on both points. First, regarding novelty: the revised manuscript no longer claims state-of-the-art predictive performance. The contribution is now explicitly framed as a systematic methodological evaluation — to our knowledge among the first to combine (i) a common 11-dimensional feature space, (ii) rigorous repeated cross-validation (5×5 = 25 evaluations), (iii) full 8-variant ablation analysis, and (iv) SHAP-based interpretability for this specific prediction task. This repositioning is stated unambiguously in the 'Main contribution' paragraph at the start of the Introduction. Second, regarding synthetic data: we fully acknowledge this limitation and have expanded the Limitations section accordingly, removing any overstatement about real-world applicability. Validation on empirical contact traces remains an explicitly stated direction for future work.

**Action taken:**

Introduction: 'Main contribution' paragraph (p. 2, lines 180–191). Section 7 Limitations: expanded (p. 13, lines 776–790).

**Response to Reviewer 2**

We thank Reviewer 2 for an exceptionally thorough and constructive review. Following these comments, we have significantly clarified the scope and positioning of the manuscript. Physics-guided neural networks are now presented explicitly as complementary, constraint-aware frameworks rather than performance-oriented alternatives — a repositioning stated in both the Introduction and the Discussion.

**Major Concern 1 — Ground-truth definition and uncertainty quantification**

**Reviewer comment:**

*The procedure is not sufficiently justified. Key details are missing — time horizon, initial conditions, absorbing states — and no uncertainty quantification is provided.*

**Response:**

Section 4.1 has been completely rewritten with a five-paragraph specification covering: (1) simulation protocol (R=200 realisations, T=80 steps, ρ₀=0.05, discrete-time SIS with synchronous update); (2) threshold identification (logistic MLE fit on a 15-point τ-grid, inflection point = τ*); (3) uncertainty quantification (σ_{τ*} median=0.0072, 95th percentile=0.0219, exclusion of networks with σ_{τ*}>0.025 — only 1 of 776 attempts); (4) absorbing state handling (realisations reaching all-susceptible state before T counted as non-outbreaks, ensuring P_out is monotone). A new 'Uncertainty estimation' paragraph explicitly states the protocol. These parameters represent a computational compromise; higher R and T would reduce σ_{τ*} further, as noted in the Limitations.

**Action taken:**

Section 4.1 (p. 6–7, lines 425–464): five-paragraph ground-truth specification. New paragraph 'Uncertainty estimation' (p. 7, lines 465–473).

**Major Concern 2 — Target leakage from β/γ as input feature**

**Reviewer comment:**

*The manuscript defines the epidemic threshold as the critical ratio β/γ, yet simultaneously includes β, γ, and β/γ among the input features. This creates ambiguity and possible partial target leakage.*

**Response:**

We thank the reviewer for identifying this important issue. The ratio β/γ has been removed from the feature vector; φ ∈ ℝ¹¹. A new paragraph 'Note on prediction task and target leakage' (Section 3.1) clarifies that the target τ* is the empirical stochastic threshold, not the analytical ratio β/γ. A simple regression τ* ~ β/γ yields R²=0.31 on our dataset, confirming that β and γ individually carry dynamical information beyond their ratio, justifying their separate inclusion. The SHAP analysis confirms this: γ (0.040) and β (0.032) rank second and third, with combined importance (0.072) exceeding any individual spectral feature.

**Action taken:**

Section 3.1 (p. 4–5, lines 324–344): φ ∈ ℝ¹¹, β/γ removed. New paragraph 'Note on prediction task and target leakage' (lines 344–353).

**Major Concern 3 — 'Physics-informed' characterisation not justified**

**Reviewer comment:**

*The characterisation as a physics-informed neural network is not convincingly supported. The constraints are heuristic and not rigorously derived from governing equations.*

**Response:**

We accept this critique fully. 'Physics-informed' has been replaced by 'physics-guided' throughout the manuscript. Section 3.2 now includes theoretical grounding for each constraint: the stability constraint derives from the QMF result (Wang et al., 2003) and the asymptotic bound of Van Mieghem & van de Bovenkamp (2015); the monotonicity constraint from spectral graph theory; the boundedness constraint from SIS dynamics. A new sentence explicitly links these constraints to the Ross–Macdonald metapopulation framework: 'Our constraints can be interpreted as low-dimensional projections of the spectral stability condition in metapopulation models.' The ablation study (Table 2) provides empirical evidence: L_bound is a genuine regulariser (+25.0% RMSE when removed), while L_stab over-regularises (−28.4%), because the QMF lower bound is violated by τ* on ≈18% of networks due to finite-size effects — violations consistent with the known asymptotic nature of QMF and not contradicting its theoretical validity.

**Action taken:**

Terminology: 'physics-guided' throughout (all sections). Section 3.2 (p. 5–6): constraint grounding + Ross–Macdonald link (lines 370–410). Table 2 (p. 10): ablation study. Section 5.2 (p. 10, lines 610–638): QMF finite-size correction.

**Major Concern 4 — Insufficient experimental evaluation**

**Reviewer comment:**

*The comparison is limited to a small set of baselines. No ablation, no statistical assessment of reported improvements.*

**Response:**

This concern has been fully addressed. Seven methods are now evaluated (QMF, KSEL, Linear Regression, Random Forest, GB, Standard MLP, PGNN). All results use 5-fold CV × 5 seeds = 25 evaluations per method (Table 1, p. 8), with mean ± std reported and Wilcoxon signed-rank tests for pairwise comparisons. Key result: GB achieves RMSE=0.0757±0.0463 (R²=0.869±0.068); PGNN shows RMSE=0.5265±0.4027 (R²=−9.138±17.553). The full 8-variant ablation study (Table 2) covers constraint ablation and feature group ablation. A new 'Statistical robustness' paragraph states the protocol explicitly. A new Section 5.2 explains the underperformance mechanistically via (i) parameter-to-sample ratio (252 for PGNN vs 88 for GB); (ii) cross-validation instability (std 8.7× higher than GB); (iii) physical constraint bias under finite-size effects.

**Action taken:**

Table 1 (p. 8): 7 methods, mean±std, 25 evaluations. Table 2 (p. 10): ablation (8 variants). Section 4.2 (p. 7): 'Statistical robustness' paragraph (lines 502–509). Section 5.2 (p. 10, lines 579–638): three-mechanism analysis.

**Major Concern 5 — Reproducibility**

**Reviewer comment:**

*Code and data should be available during the review process.*

**Response:**

The complete Python implementation (hybrid_model_complete.py, requirements.txt) and all 775 synthetic network datasets with ground-truth labels τ* are deposited on Zenodo. The DOI https://doi.org/10.5281/zenodo.19411519 will be finalised upon upload prior to resubmission; a pre-publication access link is available upon request to the corresponding author. The repository includes all code necessary to reproduce Tables 1–2, the ablation study, SHAP analysis, and KSEL calibration results, with a README verified on Python 3.10.

**Action taken:**

Declarations: 'Data and Code Availability' section updated (p. 14, lines 844–853) with Zenodo DOI.

**Journal Requirements — All 13 Points**

| **#** | **Requirement** | **Action taken** |
| --- | --- | --- |
| **1** | PLOS ONE style | Verified ✓ |
| **2** | Code sharing | Zenodo deposit (DOI: zenodo.19411519) — pre-publication access on request ✓ |
| **3** | Ethics statement | Section 4 (Methods) only; removed from other locations ✓ |
| **4** | No data restrictions | Zenodo, CC BY 4.0, unrestricted ✓ |
| **5** | GitHub 'upon acceptance' | Replaced by immediate Zenodo deposit ✓ |
| **6** | Data availability statement | Updated with Zenodo DOI (p. 14) ✓ |
| **7** | Funding Information mismatch | Financial Disclosure corrected ✓ |
| **8** | Abstract identical to form | Updated to match revised manuscript ✓ |
| **9** | Figures as separate TIFF/PNG | 4 figures uploaded as PNG 300 dpi ✓ |
| **10** | Figures 1 & 2 copyright | Replaced by author-created originals ✓ |
| **11** | Supporting Information captions | Captions at end of manuscript; in-text citations added ✓ |
| **12** | hybrid_model_complete.py unreadable | Encoding fixed (UTF-8), re-uploaded and deposited on Zenodo ✓ |
| **13** | Reviewer-suggested citations | Van Mieghem & van de Bovenkamp (2015) cited in Sections 3.2 and 5.2 ✓ |

**Summary of all changes — final revision**

**Introduction (p. 2, lines 180–191):** New 'Main contribution' paragraph: explicit repositioning as systematic methodological evaluation. Phrase 'to our knowledge' added before novelty claims.

**Section 3.1 (p. 4–5, lines 324–353):** Feature vector revised to φ ∈ ℝ¹¹ (β/γ removed). New paragraph 'Note on prediction task and target leakage' with R²=0.31 justification.

**Section 3.2 (p. 5–6, lines 370–410):** Each physical constraint theoretically grounded. New sentence linking to Ross–Macdonald spectral stability condition. 'Physics-guided' throughout.

**Section 4.1 (p. 6–7, lines 425–473):** Five-paragraph ground-truth specification (R=200, T=80, ρ₀=0.05, logistic MLE, σ_{τ*} median=0.0072). New 'Uncertainty estimation' paragraph.

**Section 4.2 (p. 7, lines 456–509):** Seven methods listed. New 'Statistical robustness' paragraph (5×5=25 evaluations, mean±std, Wilcoxon test).

**Section 5.1 — Table 1 (p. 8):** Seven methods with mean±std over 25 evaluations. Caption updated.

**Section 5.2 (p. 10, lines 579–638) — NEW:** Why PGNN underperforms: (i) parameter-to-sample ratio 252 vs 88; (ii) instability 8.7× GB; (iii) L_stab bias with QMF finite-size correction.

**Section 5.3 (p. 11, lines 640–657):** Real k* values: ER=0.787, BA=1.265, WS=0.627, REG=1.322. Corrected from original text.

**Section 5.4 (p. 11, lines 660–680):** Real SHAP values: τ_QMF=0.045 dominant, γ=0.040, β=0.032. Corrected from original text.

**Section 6 Discussion (p. 11–12):** Four insights. New subsection 6.1 'Why Physics-Guided Models Remain Relevant' (p. 12, lines 741–773): three arguments.

**Section 7 Conclusion (p. 13):** Rewritten with three-message structure. New 'Take-home message' paragraph (lines 821–829).

**Declarations (p. 14, lines 844–853):** Data/Code Availability updated with Zenodo DOI placeholder.

We are grateful for the constructive engagement of both reviewers, which has substantially improved the manuscript. We look forward to the editorial decision.

**Etienne Kouokam**

etienne.kouokam@facsciences-uy1.cm
