# Supplementary material for: A Hybrid AI-Mathematical approach for epidemic threshold prediction in metapopulation networks: Integrating physics-guided neural networks with spectral graph theory
Source: PLoS One. 2026 Jun 18;21(6):e0344827. doi: 10.1371/journal.pone.0344827 (PMC13278587; doi:10.1371/journal.pone.0344827)
Supplement: Attachment — Submitted filename: Response_to_Reviewers_PONE_R3.docx [file pone.0344827.s004.docx]

**Response to Reviewers — Third Round**

**Manuscript:** PONE-D-26-09719R1

**Title:** *A Hybrid AI-Mathematical Approach for Epidemic Threshold Prediction in Metapopulation Networks: Integrating Physics-Guided Neural Networks with Spectral Graph Theory*

**Journal:** PLOS ONE

**Authors:** Etienne Kouokam

**Date:** May 2026

**General Response**

We thank the Academic Editor (Prof. Americo Cunha Jr) and both reviewers for their careful reading of the revised manuscript and their constructive assessment. We are gratified that Reviewer #1 finds all previous comments adequately addressed and considers the manuscript acceptable for publication. We have carefully studied the four remaining minor points raised by Reviewer #2 and have addressed each of them through targeted additions to the manuscript text. We believe these clarifications further strengthen the scientific precision and balance of the paper without altering its core contributions. A detailed point-by-point response follows below.

**Reviewer #1**

*“The manuscript presents a technically sound and reproducible comparative study and addressed previous comments. The experimental evaluation updated and is thorough and transparent, particularly the uncertainty analysis, ablation study, and public code/data availability and author also clarify it’s litigation while doing this study.”*

We thank Reviewer #1 for this positive assessment and for confirming that all previous comments have been addressed. No further changes were requested; we have nonetheless ensured that the additional clarifications introduced in response to Reviewer #2 remain fully consistent with the points validated in this round.

**Reviewer #2**

Reviewer #2 acknowledges substantial improvement in the manuscript and raises four minor points for further clarification. We address each point below in the order presented.

**Point 1 — Robustness and sensitivity of the epidemic threshold criterion**

*“The definition of the epidemic threshold, while now clearly specified, remains based on a heuristic transition criterion, and the manuscript would be strengthened by a brief discussion of its robustness or sensitivity.”*

We agree that this point deserves explicit discussion. The epidemic threshold is estimated via the susceptibility-peak criterion χ(β) = n·Var(ρ)/(ρ̄ + ε), which is a widely used operational estimator in the computational epidemiology literature (Chakrabarti et al., 2008; Pastor-Satorras et al., 2015). As already noted in the manuscript (Section 4.3), N_mc = 20 Monte Carlo replicates and N_β = 25 grid points were used. The robustness analysis over μ ∈ {0.3, 0.5, 0.7} (Section 5.5, Figure 5b) implicitly assesses sensitivity to this estimator, since varying μ shifts the location of the threshold along the β axis. We have added a concise paragraph in Section 4.3 acknowledging the heuristic nature of the susceptibility-peak estimator and its known sensitivity in specific regimes (e.g., strongly periodic networks), referencing the PRIMARY SCHOOL case discussed in Section 7.4 as a concrete illustration. We also note that a multi-criteria comparison (persistence time, final epidemic size, branching factor) is an important direction for future work, as now stated explicitly in the Limitations paragraph.

**Manuscript change:** *Added ∼3 sentences to Section 4.3 (after Eq. 1) discussing the robustness of the susceptibility-peak estimator and its sensitivity in strongly periodic networks, with a forward reference to Section 7.4 (PRIMARY SCHOOL). Added one sentence to Section 8 (Limitations) on multi-criteria threshold estimation as future work.*

**Point 2 — Use of τ_QMF as an input feature versus benchmark**

*“The use of τ_QMF as an input feature, while now acknowledged, continues to raise some interpretational questions in relation to the benchmarking against QMF itself, and this point could be clarified further.”*

We thank the reviewer for pressing on this point. The potential for circular reasoning is a legitimate concern and deserves a clearer explanation. The key clarification is as follows: τ_QMF = 1/ρ(Ā̅) is used as one of the input features to the physics-guided neural network because it encodes a theoretically motivated baseline (the mean-field approximation), not because it already provides an accurate estimate of λ_c. The benchmarking comparison is between (i) the raw QMF estimate τ_QMF used as a standalone predictor and (ii) our neural network that takes τ_QMF among other spectral features as input and outputs a corrected estimate. The improvement thus measures how much the network learns to correct the systematic bias of the QMF approximation using additional structural information. This is analogous to residual learning in physics-informed machine learning: the model learns the deviation from a known imperfect baseline. We have added an explicit clarifying note in Section 3 (Feature Design) to make this distinction unambiguous for the reader.

**Manuscript change:** *Added a clarifying note (∼4 sentences) in Section 3 explaining that τ_QMF serves as a physics-motivated input feature encoding the mean-field baseline, and that the benchmarking evaluates the network’s capacity to correct this baseline—not a circular comparison. Analogy to residual learning made explicit.*

**Point 3 — Scope of validity of the physics-guided constraints**

*“The constraints introduced in the model, although useful as regularizers, remain only loosely connected to formal theoretical derivations, and their scope of validity could be discussed more explicitly.”*

We agree with the reviewer that the theoretical grounding of the physics-guided constraints merits more explicit treatment. The constraints encode two well-established properties of the epidemic threshold: (C1) monotone decrease with network connectivity (captured by the positivity constraint on the spectral-radius coefficient), and (C2) positivity of predictions (guaranteed by log-space parameterisation). Both are direct consequences of the QMF approximation λ_c ∼ μ/ρ(Ā̅) under the assumptions of the mean-field model (homogeneous recovery, Markovian SIS dynamics). We now explicitly state in Section 3 that these constraints are valid within the regime of applicability of the QMF approximation—namely, networks with moderate temporal heterogeneity (σ²_λ/λ̅²₁ < 0.5, as defined in Section 6)—and may lose theoretical motivation for strongly heterogeneous networks (e.g., activity-driven configurations), where they act primarily as regularizers ensuring numerical stability and output positivity.

**Manuscript change:** *Added ∼3 sentences to Section 3 (Physics-Guided Constraints) clarifying: (a) the theoretical basis of each constraint within the QMF regime; (b) the regime of validity (σ²/λ̅² < 0.5); (c) their role as regularizers outside this regime. Cross-reference to Theorem 6.1 and Remark 6.2 added.*

**Point 4 — Scope of applicability claims**

*“The study is still largely restricted to synthetic networks, and although this limitation is recognized, the claims regarding applicability should remain cautious in the absence of validation on empirical data.”*

We fully agree. While Section 7 provides empirical validation on four SocioPatterns real-world contact networks, the neural network itself was trained exclusively on synthetic data, and the empirical results are presented as out-of-distribution generalization tests rather than a comprehensive validation. We have revisited the Abstract, Introduction (end of Section 1.2), and Discussion (Section 8) to replace unqualified applicability claims with appropriately hedged formulations. Specifically, phrases of the form “directly applicable to data from hospitals, schools, and conferences” have been revised to “potentially applicable, subject to empirical validation on data from [context]”, and the in-regime/out-of-regime distinction is now referenced in these contexts.

**Manuscript change:** *Revised 3 sentences in the Abstract, Section 1.2, and Section 8 to replace unqualified applicability claims with hedged formulations referencing the in-regime condition (σ²/λ̅² < 0.5) and the need for further empirical validation. No structural or methodological changes.*

**Summary of Changes**

The table below summarises all modifications made to the manuscript in this revision round.

| **#** | **Reviewer concern** | **Change made** | **Location** |
| --- | --- | --- | --- |
| **1** | Robustness of threshold criterion | Added ∼3 sentences on sensitivity of susceptibility-peak estimator; future work on multi-criteria estimation | *Sec. 4.3, Sec. 8* |
| **2** | τ_QMF as feature vs benchmark | Added clarification: τ_QMF encodes the mean-field baseline; network learns to correct it (residual learning analogy) | *Sec. 3* |
| **3** | Scope of physics-guided constraints | Added theoretical basis, regime of validity (σ²/λ̅² < 0.5), and regularizer role outside regime | *Sec. 3, cross-ref Thm 6.1* |
| **4** | Applicability claims to empirical data | Revised 3 sentences: unqualified claims → hedged formulations referencing in-regime condition | *Abstract, Sec. 1.2, Sec. 8* |

**Closing Statement**

We believe that the revised manuscript fully addresses the minor concerns raised in this round. The core contributions—the physics-guided neural network architecture, the rigorous evaluation protocol (LOO-CV, bootstrap CIs, ablation study), and the public code/data availability—remain unchanged. We are confident that the manuscript is now ready for publication in PLOS ONE and hope that the Editor and Reviewer #2 will find the revised version satisfactory.

We thank the editorial team for their continued support of this work.

Sincerely,

**Dr. Etienne Kouokam**

Université de Yaoundé I / IRD-UMMISCO, Sorbonne Université

*etienne.kouokam@facsciences-uy1.cm*
