# Supplementary material for: A Hybrid AI-Mathematical approach for epidemic threshold prediction in metapopulation networks: Integrating physics-guided neural networks with spectral graph theory
Source: PLoS One. 2026 Jun 18;21(6):e0344827. doi: 10.1371/journal.pone.0344827 (PMC13278587; doi:10.1371/journal.pone.0344827)
Supplement: Peer review history

# Peer review: Decision Letter 0

**DOI:** 10.1371/journal.pone.0344827.r001

**Authors:** Americo Cunha

31 Mar 2026

PONE-D-26-09719

A Hybrid AI-Mathematical Approach for Epidemic Threshold Prediction in Metapopulation Networks: Integrating Physics-Informed Neural Networks with Spectral Graph Theory

PLOS One

Dear Dr. Kouokam,

Thank you for submitting your manuscript to PLOS ONE. After careful consideration, we feel that it has merit but does not fully meet PLOS ONE’s publication criteria as it currently stands. Therefore, we invite you to submit a revised version of the manuscript that addresses the points raised during the review process.

==============================

The manuscript still requires substantial revision before it can be considered for publication. In particular, you must ensure that the methodological foundations of the study are clearly defined and rigorously justified. The definition of the epidemic threshold used as ground truth needs to be explained in detail and supported with appropriate justification, including how it is computed and how uncertainty is handled. In addition, the formulation of the learning problem must be clarified to avoid ambiguity and potential target leakage, especially regarding the use of input features that are directly related to the predicted quantity.

You should also revisit the characterization of the model as “physics-informed,” ensuring that this claim is either rigorously supported by theory or appropriately moderated. The experimental validation must be strengthened by including additional baseline methods, ablation analyses, and, where possible, statistical assessment of performance. Furthermore, the scope of the study should be reconsidered, as validation on real-world data or a more cautious interpretation of applicability is necessary.

Finally, to improve transparency and reproducibility, the code and datasets should be made available during the review process. Addressing these points is essential for the manuscript to meet the standards required for publication.

==============================

Please submit your revised manuscript by May 15 2026 11:59PM. If you will need more time than this to complete your revisions, please reply to this message or contact the journal office at plosone@plos.org. When you're ready to submit your revision, log on to https://www.editorialmanager.com/pone/ and select the 'Submissions Needing Revision' folder to locate your manuscript file.

Please include the following items when submitting your revised manuscript:

- A letter that responds to each point raised by the academic editor and reviewer(s). You should upload this letter as a separate file labeled 'Response to Reviewers'.
- A marked-up copy of your manuscript that highlights changes made to the original version. You should upload this as a separate file labeled 'Revised Manuscript with Track Changes'.
- An unmarked version of your revised paper without tracked changes. You should upload this as a separate file labeled 'Manuscript'.

If you would like to make changes to your financial disclosure, please include your updated statement in your cover letter. Guidelines for resubmitting your figure files are available below the reviewer comments at the end of this letter.

If applicable, we recommend that you deposit your laboratory protocols in protocols.io to enhance the reproducibility of your results. Protocols.io assigns your protocol its own identifier (DOI) so that it can be cited independently in the future. For instructions see: https://journals.plos.org/plosone/s/submission-guidelines#loc-laboratory-protocols. Additionally, PLOS ONE offers an option for publishing peer-reviewed Lab Protocol articles, which describe protocols hosted on protocols.io. Read more information on sharing protocols at https://plos.org/protocols?utm_medium=editorial-email&utm_source=authorletters&utm_campaign=protocols.

As the corresponding author, your ORCID iD is verified in the submission system and will appear in the published article. PLOS supports the use of ORCID, and we encourage all coauthors to register for an ORCID iD and use it as well. Please encourage your coauthors to verify their ORCID iD within the submission system before final acceptance, as unverified ORCID iDs will not appear in the published article. *Only*     the individual author can complete the verification step; PLOS staff *cannot*     verify ORCID iDs on behalf of authors.

We look forward to receiving your revised manuscript.

Kind regards,

Americo Cunha Jr

Academic Editor

PLOS One

Journal Requirements:

When submitting your revision, we need you to address these additional requirements.

1. Please ensure that your manuscript meets PLOS ONE's style requirements, including those for file naming. The PLOS ONE style templates can be found at

https://journals.plos.org/plosone/s/file?id=wjVg/PLOSOne_formatting_sample_main_body.pdf and https://journals.plos.org/plosone/s/file?id=ba62/PLOSOne_formatting_sample_title_authors_affiliations.pdf

2. Please note that PLOS One has specific guidelines on code sharing for submissions in which author-generated code underpins the findings in the manuscript. In these cases, we expect all author-generated code to be made available without restrictions upon publication of the work. Please review our guidelines at https://journals.plos.org/plosone/s/materials-and-software-sharing#loc-sharing-code and ensure that your code is shared in a way that follows best practice and facilitates reproducibility and reuse.

3. Your ethics statement should only appear in the Methods section of your manuscript. If your ethics statement is written in any section besides the Methods, please move it to the Methods section and delete it from any other section. Please ensure that your ethics statement is included in your manuscript, as the ethics statement entered into the online submission form will not be published alongside your manuscript.

4. We note that you have indicated that there are restrictions to data sharing for this study. PLOS only allows data to be available upon request if there are legal or ethical restrictions on sharing data publicly. For more information on unacceptable data access restrictions, please see http://journals.plos.org/plosone/s/data-availability#loc-unacceptable-data-access-restrictions.

Before we proceed with your manuscript, please address the following prompts:

a) If there are ethical or legal restrictions on sharing a de-identified data set, please explain them in detail (e.g., data contain potentially identifying or sensitive patient information, data are owned by a third-party organization, etc.) and who has imposed them (e.g., a Research Ethics Committee or Institutional Review Board, etc.). Please also provide contact information for a data access committee, ethics committee, or other institutional body to which data requests may be sent.

b) If there are no restrictions, please upload the minimal anonymized data set necessary to replicate your study findings to a stable, public repository and provide us with the relevant URLs, DOIs, or accession numbers. For a list of recommended repositories, please see

https://journals.plos.org/plosone/s/recommended-repositories. You also have the option of uploading the data as Supporting Information files, but we would recommend depositing data directly to a data repository if possible.

We will update your Data Availability statement on your behalf to reflect the information you provide.

5. In the online submission form, you indicated that the complete Python implementation and all synthetic network datasets underlying the findings will be deposited in a public GitHub repository upon acceptance of the manuscript. Requests for access prior to publication may be directed to the corresponding author at etienne.kouokam@facsciences-uy1.cm.

All PLOS journals now require all data underlying the findings described in their manuscript to be freely available to other researchers, either 1. In a public repository, 2. Within the manuscript itself, or 3. Uploaded as supplementary information.

This policy applies to all data except where public deposition would breach compliance with the protocol approved by your research ethics board. If your data cannot be made publicly available for ethical or legal reasons (e.g., public availability would compromise patient privacy), please explain your reasons on resubmission and your exemption request will be escalated for approval.

6. When completing the data availability statement of the submission form, you indicated that you will make your data available on acceptance. We strongly recommend all authors decide on a data sharing plan before acceptance, as the process can be lengthy and hold up publication timelines. Please note that, though access restrictions are acceptable now, your entire data will need to be made freely accessible if your manuscript is accepted for publication. This policy applies to all data except where public deposition would breach compliance with the protocol approved by your research ethics board. If you are unable to adhere to our open data policy, please kindly revise your statement to explain your reasoning and we will seek the editor's input on an exemption. Please be assured that, once you have provided your new statement, the assessment of your exemption will not hold up the peer review process.

7. We note that the grant information you provided in the ‘Funding Information’ and ‘Financial Disclosure’ sections do not match.

When you resubmit, please ensure that you provide the correct grant numbers for the awards you received for your study in the ‘Funding Information’ section.

8. Please amend either the abstract on the online submission form (via Edit Submission) or the abstract in the manuscript so that they are identical.

9. Please remove your figures from within your manuscript file, leaving only the individual TIFF/EPS image files, uploaded separately. These will be automatically included in the reviewers’ PDF**.**

10. We note that Figure(s) 1, 2 in your submission contain copyrighted images. All PLOS content is published under the Creative Commons Attribution License (CC BY 4.0), which means that the manuscript, images, and Supporting Information files will be freely available online, and any third party is permitted to access, download, copy, distribute, and use these materials in any way, even commercially, with proper attribution. For more information, see our copyright guidelines: http://journals.plos.org/plosone/s/licenses-and-copyright.

We require you to either (1) present written permission from the copyright holder to publish these figures specifically under the CC BY 4.0 license, or (2) remove the figures from your submission:

a. You may seek permission from the original copyright holder of Figure(s) 1, 2 to publish the content specifically under the CC BY 4.0 license.

We recommend that you contact the original copyright holder with the Content Permission Form (http://journals.plos.org/plosone/s/file?id=7c09/content-permission-form.pdf) and the following text:

“I request permission for the open-access journal PLOS ONE to publish XXX under the Creative Commons Attribution License (CCAL) CC BY 4.0 (http://creativecommons.org/licenses/by/4.0/). Please be aware that this license allows unrestricted use and distribution, even commercially, by third parties. Please reply and provide explicit written permission to publish XXX under a CC BY license and complete the attached form.”

Please upload the completed Content Permission Form or other proof of granted permissions as an "Other" file with your submission.

In the figure caption of the copyrighted figure, please include the following text: “Reprinted from [ref] under a CC BY license, with permission from [name of publisher], original copyright [original copyright year].”

b. If you are unable to obtain permission from the original copyright holder to publish these figures under the CC BY 4.0 license or if the copyright holder’s requirements are incompatible with the CC BY 4.0 license, please either i) remove the figure or ii) supply a replacement figure that complies with the CC BY 4.0 license. Please check copyright information on all replacement figures and update the figure caption with source information. If applicable, please specify in the figure caption text when a figure is similar but not identical to the original image and is therefore for illustrative purposes only.

11. Please include captions for your Supporting Information files at the end of your manuscript, and update any in-text citations to match accordingly. Please see our Supporting Information guidelines for more information: http://journals.plos.org/plosone/s/supporting-information.

12. We are unable to open your Supporting Information file [hybrid_model_complete.py]. Please kindly revise as necessary and re-upload.

13. If the reviewer comments include a recommendation to cite specific previously published works, please review and evaluate these publications to determine whether they are relevant and should be cited. There is no requirement to cite these works unless the editor has indicated otherwise.

Additional Editor Comments :

The manuscript still requires substantial revision before it can be considered for publication. In particular, you must ensure that the methodological foundations of the study are clearly defined and rigorously justified. The definition of the epidemic threshold used as ground truth needs to be explained in detail and supported with appropriate justification, including how it is computed and how uncertainty is handled. In addition, the formulation of the learning problem must be clarified to avoid ambiguity and potential target leakage, especially regarding the use of input features that are directly related to the predicted quantity.

You should also revisit the characterization of the model as “physics-informed,” ensuring that this claim is either rigorously supported by theory or appropriately moderated. The experimental validation must be strengthened by including additional baseline methods, ablation analyses, and, where possible, statistical assessment of performance. Furthermore, the scope of the study should be reconsidered, as validation on real-world data or a more cautious interpretation of applicability is necessary.

Finally, to improve transparency and reproducibility, the code and datasets should be made available during the review process. Addressing these points is essential for the manuscript to meet the standards required for publication.

[Note: HTML markup is below. Please do not edit.]

Reviewers' comments:

Reviewer's Responses to Questions

**Comments to the Author**

1. Is the manuscript technically sound, and do the data support the conclusions?

The manuscript must describe a technically sound piece of scientific research with data that supports the conclusions. Experiments must have been conducted rigorously, with appropriate controls, replication, and sample sizes. The conclusions must be drawn appropriately based on the data presented.

Reviewer #1: Yes

Reviewer #2: No

**********

2. Has the statistical analysis been performed appropriately and rigorously?

Reviewer #1: Yes

Reviewer #2: No

**********

3. Have the authors made all data underlying the findings in their manuscript fully available?

The PLOS Data policy requires authors to make all data underlying the findings described in their manuscript fully available without restriction, with rare exception (please refer to the Data Availability Statement in the manuscript PDF file). The data should be provided as part of the manuscript or its supporting information, or deposited to a public repository. For example, in addition to summary statistics, the data points behind means, medians and variance measures should be available. If there are restrictions on publicly sharing data—e.g. participant privacy or use of data from a third party—those must be specified.

Reviewer #1: Yes

Reviewer #2: No

**********

4. Is the manuscript presented in an intelligible fashion and written in standard English?

PLOS ONE does not copyedit accepted manuscripts, so the language in submitted articles must be clear, correct, and unambiguous. Any typographical or grammatical errors should be corrected at revision, so please note any specific errors here.

Reviewer #1: Yes

Reviewer #2: Yes

**********

5. Review Comments to the Author

Please use the space provided to explain your answers to the questions above. You may also include additional comments for the author, including concerns about dual publication, research ethics, or publication ethics. (Please upload your review as an attachment if it exceeds 20,000 characters)

Reviewer #1: The manuscript presents a technically sound and well-structured hybrid framework integrating physics-informed neural networks with spectral graph theory for epidemic threshold prediction. The methodology is appropriate and experiments are conducted on a reasonably diverse set of synthetic networks, showing clear improvements over established baselines. The inclusion of interpretability analysis further strengthens the practical relevance of the work.

However, the novelty is somewhat incremental, and the evaluation relies entirely on synthetic data with simulation-based ground truth, limiting evidence of real-world applicability. The manuscript would benefit from validation on empirical contact network data and discussion of generalization to real-world scenarios.

Overall, the work meets the methodological and reproducibility standards of PLOS ONE, and I recommend acceptance and a minor revisions addressing the above points will benefits the work more

Reviewer #2: This manuscript addresses an important problem in computational epidemiology, namely the prediction of epidemic thresholds in complex networks, and proposes a hybrid approach combining spectral graph features with a neural model described as physics-informed. The topic is appropriate for a multidisciplinary journal such as PLOS ONE, and the paper is generally well structured, clearly written, and supported by a coherent experimental pipeline. However, in its current form, the manuscript does not yet meet the level of methodological rigor and technical soundness required for publication, and substantial revisions are necessary before it can be considered suitable.

The most critical issue concerns the definition of the target variable used throughout the study. The “true” epidemic threshold is obtained via stochastic SIS simulations using a heuristic criterion based on a transition in outbreak probability between 10% and 90%. This procedure is not sufficiently justified, and key details are missing, including how the threshold is numerically identified, the time horizon of simulations, the role of initial conditions, and the handling of absorbing states. Moreover, no uncertainty quantification is provided, despite the acknowledged presence of Monte Carlo noise. Since all reported performance metrics depend on this estimated ground truth, the lack of a rigorous and validated definition undermines the credibility of the results and constitutes a major obstacle to publication.

A second major concern is the conceptual formulation of the learning problem. The manuscript defines the epidemic threshold as the critical ratio β/γ, yet simultaneously includes β, γ, and β/γ among the input features used to predict that same quantity. This creates ambiguity and raises the possibility of redundancy or partial target leakage. The paper does not clearly distinguish between the parameters used to generate simulations and the quantity being predicted, and this lack of clarity weakens both the interpretation of the model and the validity of the comparisons. A precise and consistent formulation of the prediction task is required.

The characterization of the method as a physics-informed neural network is also not convincingly supported. The constraints incorporated into the loss function are heuristic (monotonicity, boundedness, and a relation to the spectral radius) and are not rigorously derived from the governing equations of the Ross–Macdonald or related epidemic models. While these constraints may act as useful regularizers, the connection to underlying epidemiological theory is largely qualitative rather than formal. As a result, the current presentation overstates the theoretical grounding of the approach. Either the constraints should be derived more rigorously, or the claims regarding “physics-informed” modeling should be significantly moderated.

The experimental validation is also insufficient for a study of this type. The comparison is limited to a small set of baselines, and important alternatives—such as tree-based regression methods or other nonparametric models using the same feature set—are not considered. There is no ablation analysis to assess the contribution of individual features or constraints, and no statistical assessment of the reported improvements. In addition, the model uses τ_QMF as an input feature while being compared against QMF itself, which requires careful interpretation and explicit discussion, as it effectively embeds one of the baselines into the proposed method. Without a more comprehensive and carefully controlled benchmarking strategy, the reported performance gains are difficult to evaluate.

The scope of the study is further limited by its exclusive reliance on synthetic networks. Although the manuscript acknowledges this limitation, the absence of validation on empirical contact networks significantly reduces the practical relevance of the results, particularly given the strong claims regarding applications to real-time surveillance and public health decision-making. At a minimum, the claims about applicability should be tempered; ideally, the study should include at least one real dataset or a more detailed discussion of generalization beyond the synthetic setting.

Reproducibility is another concern. The manuscript states that code and data will be made available upon acceptance, but for a computational study of this nature, availability during the review process is highly desirable and often expected. Making the implementation and datasets accessible at submission would substantially strengthen the paper.

Finally, the manuscript would benefit from a broader and deeper engagement with the existing literature. The number of references is limited relative to the scope of the topic, and the positioning of the work within current research on epidemic processes on networks, machine learning surrogates, and physics-informed modeling remains somewhat narrow.

In summary, while the paper addresses a relevant problem and presents an interesting combination of ideas, it currently falls short of PLOS ONE’s standards for technical soundness and completeness. To become suitable for publication, the authors should provide a rigorous and well-justified definition of the epidemic threshold used as ground truth, clarify the formulation of the learning problem to avoid ambiguity or leakage, either substantiate or moderate the claims regarding physics-informed modeling, substantially strengthen the experimental evaluation with additional baselines and ablations, improve reproducibility by releasing code and data, and, if possible, include validation on empirical networks or temper claims accordingly. With these improvements, the manuscript could evolve into a solid and publishable contribution.

**********

6. PLOS authors have the option to publish the peer review history of their article (what does this mean?). If published, this will include your full peer review and any attached files.

If you choose “no”, your identity will remain anonymous but your review may still be made public.

**Do you want your identity to be public for this peer review?**     For information about this choice, including consent withdrawal, please see our Privacy Policy.

Reviewer #1: No

Reviewer #2: No

**********

[NOTE: If reviewer comments were submitted as an attachment file, they will be attached to this email and accessible via the submission site. Please log into your account, locate the manuscript record, and check for the action link "View Attachments". If this link does not appear, there are no attachment files.]

To ensure your figures meet our technical requirements, please review our figure guidelines: https://journals.plos.org/plosone/s/figures

You may also use PLOS’s free figure tool, NAAS, to help you prepare publication quality figures: https://journals.plos.org/plosone/s/figures#loc-tools-for-figure-preparation.

NAAS will assess whether your figures meet our technical requirements by comparing each figure against our figure specifications.

# Author comment: Author response to Decision Letter 1

**DOI:** 10.1371/journal.pone.0344827.r002

24 Apr 2026

We thank the Academic Editor and both reviewers for their constructive comments. All five major concerns raised by Reviewer 2 have been fully addressed in the revised manuscript:

(1) Ground-truth definition and uncertainty quantification: Section 4.1 fully rewritten with complete protocol specification and new "Uncertainty estimation" paragraph (p. 7).

(2) Target leakage: β/γ removed from feature vector; φ ∈ ℝ¹¹; clarification paragraph added (Section 3.1, p. 4–5).

(3) Physics-guided terminology: "physics-informed" replaced by "physics-guided" throughout; constraints theoretically grounded (Section 3.2).

(4) Experimental evaluation: 7 methods, 5-fold CV × 5 seeds = 25 evaluations, mean ± std, full ablation study (Table 2), new Section 5.2.

(5) Reproducibility: complete code and data publicly available at https://doi.org/10.5281/zenodo.19411519

A detailed point-by-point response is provided in the attached "Response to Reviewers" document.

Attachment Submitted filename: Response_to_Reviewers_R2_Final_PONE_D_26_09719_v2.docx

# Peer review: Decision Letter 1

**DOI:** 10.1371/journal.pone.0344827.r003

**Authors:** Americo Cunha

21 May 2026

PONE-D-26-09719R1

A Hybrid AI-Mathematical Approach for Epidemic Threshold Prediction in Metapopulation Networks: .Integrating Physics-Guided Neural Networks with Spectral Graph Theory

PLOS One

Dear Dr. Kouokam,

Thank you for submitting your manuscript to PLOS ONE. After careful consideration, we feel that it has merit but does not fully meet PLOS ONE’s publication criteria as it currently stands. Therefore, we invite you to submit a revised version of the manuscript that addresses the points raised during the review process.

==============================

**Take into account the minor changes asked by referee 1.**

==============================

Please submit your revised manuscript by Jul 05 2026 11:59PM. If you will need more time than this to complete your revisions, please reply to this message or contact the journal office at plosone@plos.org. When you're ready to submit your revision, log on to https://www.editorialmanager.com/pone/ and select the 'Submissions Needing Revision' folder to locate your manuscript file.

Please include the following items when submitting your revised manuscript:

- A letter that responds to each point raised by the academic editor and reviewer(s). You should upload this letter as a separate file labeled 'Response to Reviewers'.
- A marked-up copy of your manuscript that highlights changes made to the original version. You should upload this as a separate file labeled 'Revised Manuscript with Track Changes'.
- An unmarked version of your revised paper without tracked changes. You should upload this as a separate file labeled 'Manuscript'.

If you would like to make changes to your financial disclosure, please include your updated statement in your cover letter. Guidelines for resubmitting your figure files are available below the reviewer comments at the end of this letter.

If applicable, we recommend that you deposit your laboratory protocols in protocols.io to enhance the reproducibility of your results. Protocols.io assigns your protocol its own identifier (DOI) so that it can be cited independently in the future. For instructions see: https://journals.plos.org/plosone/s/submission-guidelines#loc-laboratory-protocols. Additionally, PLOS ONE offers an option for publishing peer-reviewed Lab Protocol articles, which describe protocols hosted on protocols.io. Read more information on sharing protocols at https://plos.org/protocols?utm_medium=editorial-email&utm_source=authorletters&utm_campaign=protocols.

As the corresponding author, your ORCID iD is verified in the submission system and will appear in the published article. PLOS supports the use of ORCID, and we encourage all coauthors to register for an ORCID iD and use it as well. Please encourage your coauthors to verify their ORCID iD within the submission system before final acceptance, as unverified ORCID iDs will not appear in the published article. *Only*     the individual author can complete the verification step; PLOS staff *cannot*     verify ORCID iDs on behalf of authors.

We look forward to receiving your revised manuscript.

Kind regards,

Americo Cunha Jr

Academic Editor

PLOS One

Journal Requirements:

1. If the reviewer comments include a recommendation to cite specific previously published works, please review and evaluate these publications to determine whether they are relevant and should be cited. There is no requirement to cite these works unless the editor has indicated otherwise.

2. Please review your reference list to ensure that it is complete and correct. If you have cited papers that have been retracted, please include the rationale for doing so in the manuscript text, or remove these references and replace them with relevant current references. Any changes to the reference list should be mentioned in the rebuttal letter that accompanies your revised manuscript. If you need to cite a retracted article, indicate the article’s retracted status in the References list and also include a citation and full reference for the retraction notice.

**Additional Editor Comments:**

Please take into account the minor comments.

[Note: HTML markup is below. Please do not edit.]

Reviewers' comments:

Reviewer's Responses to Questions

**Comments to the Author**

1. If the authors have adequately addressed your comments raised in a previous round of review and you feel that this manuscript is now acceptable for publication, you may indicate that here to bypass the “Comments to the Author” section, enter your conflict of interest statement in the “Confidential to Editor” section, and submit your "Accept" recommendation.

Reviewer #1: All comments have been addressed

Reviewer #2: (No Response)

**********

2. Is the manuscript technically sound, and do the data support the conclusions?

The manuscript must describe a technically sound piece of scientific research with data that supports the conclusions. Experiments must have been conducted rigorously, with appropriate controls, replication, and sample sizes. The conclusions must be drawn appropriately based on the data presented.

Reviewer #1: Yes

Reviewer #2: Yes

**********

3. Has the statistical analysis been performed appropriately and rigorously?

Reviewer #1: Yes

Reviewer #2: Yes

**********

4. Have the authors made all data underlying the findings in their manuscript fully available?

The PLOS Data policy requires authors to make all data underlying the findings described in their manuscript fully available without restriction, with rare exception (please refer to the Data Availability Statement in the manuscript PDF file). The data should be provided as part of the manuscript or its supporting information, or deposited to a public repository. For example, in addition to summary statistics, the data points behind means, medians and variance measures should be available. If there are restrictions on publicly sharing data—e.g. participant privacy or use of data from a third party—those must be specified.

Reviewer #1: Yes

Reviewer #2: Yes

**********

5. Is the manuscript presented in an intelligible fashion and written in standard English?

PLOS ONE does not copyedit accepted manuscripts, so the language in submitted articles must be clear, correct, and unambiguous. Any typographical or grammatical errors should be corrected at revision, so please note any specific errors here.

Reviewer #1: Yes

Reviewer #2: Yes

**********

6. Review Comments to the Author

Please use the space provided to explain your answers to the questions above. You may also include additional comments for the author, including concerns about dual publication, research ethics, or publication ethics. (Please upload your review as an attachment if it exceeds 20,000 characters)

Reviewer #1: The manuscript presents a technically sound and reproducible comparative study and addressed previous commentss. The experimental evaluation updated and is thorough and transparent, particularly the uncertainty analysis, ablation study, and public code/data availability and author also clarify it's litigation while doing this study.

Reviewer #2: The revised manuscript shows clear and substantive improvement compared to the previous version. The author has taken the earlier comments seriously and has strengthened the work in several important respects. In particular, the definition of the ground-truth epidemic threshold is now much better documented, with a transparent simulation protocol and the addition of Monte Carlo–based uncertainty quantification, which significantly enhances the methodological credibility of the study. The formulation of the learning problem has also been clarified, notably through the removal of the β/γ ratio from the feature set and a clearer distinction between input parameters and the prediction target, resolving the earlier concern about potential leakage and ambiguity.

The repositioning of the method as “physics-guided” rather than “physics-informed” is appropriate and reflects a more accurate interpretation of the role of the constraints. More broadly, the author has improved the scientific balance of the manuscript by moderating the claims and explicitly acknowledging the limitations of the proposed model. The experimental section has been substantially strengthened, with the inclusion of additional baselines, repeated cross-validation, statistical assessment, and a thorough ablation study. The decision to make code and data publicly available is also a very positive development that greatly improves reproducibility.

Despite these improvements, a few aspects would still benefit from further refinement. The definition of the epidemic threshold, while now clearly specified, remains based on a heuristic transition criterion, and the manuscript would be strengthened by a brief discussion of its robustness or sensitivity. The use of τ_QMF as an input feature, while now acknowledged, continues to raise some interpretational questions in relation to the benchmarking against QMF itself, and this point could be clarified further. In addition, the constraints introduced in the model, although useful as regularizers, remain only loosely connected to formal theoretical derivations, and their scope of validity could be discussed more explicitly. Finally, the study is still largely restricted to synthetic networks, and although this limitation is recognized, the claims regarding applicability should remain cautious in the absence of validation on empirical data.

Overall, the manuscript is now much more solid, transparent, and scientifically balanced. The main concerns raised in the initial review have been largely addressed, and the remaining issues are relatively minor and do not undermine the technical soundness of the work.

**********

7. PLOS authors have the option to publish the peer review history of their article (what does this mean?). If published, this will include your full peer review and any attached files.

If you choose “no”, your identity will remain anonymous but your review may still be made public.

**Do you want your identity to be public for this peer review?**     For information about this choice, including consent withdrawal, please see our Privacy Policy.

Reviewer #1: No

Reviewer #2: No

**********

[NOTE: If reviewer comments were submitted as an attachment file, they will be attached to this email and accessible via the submission site. Please log into your account, locate the manuscript record, and check for the action link "View Attachments". If this link does not appear, there are no attachment files.]

To ensure your figures meet our technical requirements, please review our figure guidelines: https://journals.plos.org/plosone/s/figures

You may also use PLOS’s free figure tool, NAAS, to help you prepare publication quality figures: https://journals.plos.org/plosone/s/figures#loc-tools-for-figure-preparation.

NAAS will assess whether your figures meet our technical requirements by comparing each figure against our figure specifications.

# Author comment: Author response to Decision Letter 2

**DOI:** 10.1371/journal.pone.0344827.r004

26 May 2026

Response to Reviewers — Third Round

Manuscript: PONE-D-26-09719R1

Title: A Hybrid AI-Mathematical Approach for Epidemic Threshold Prediction in Metapopulation Networks: Integrating Physics-Guided Neural Networks with Spectral Graph Theory

Journal: PLOS ONE

Authors: Etienne Kouokam

Date: May 2026

General Response

We thank the Academic Editor (Prof. Americo Cunha Jr) and both reviewers for their careful reading of the revised manuscript and their constructive assessment. We are gratified that Reviewer #1 finds all previous comments adequately addressed and considers the manuscript acceptable for publication. We have carefully studied the four remaining minor points raised by Reviewer #2 and have addressed each of them through targeted additions to the manuscript text. We believe these clarifications further strengthen the scientific precision and balance of the paper without altering its core contributions. A detailed point-by-point response follows below.

Reviewer #1

“The manuscript presents a technically sound and reproducible comparative study and addressed previous comments. The experimental evaluation updated and is thorough and transparent, particularly the uncertainty analysis, ablation study, and public code/data availability and author also clarify it’s litigation while doing this study.”

We thank Reviewer #1 for this positive assessment and for confirming that all previous comments have been addressed. No further changes were requested; we have nonetheless ensured that the additional clarifications introduced in response to Reviewer #2 remain fully consistent with the points validated in this round.

Reviewer #2

Reviewer #2 acknowledges substantial improvement in the manuscript and raises four minor points for further clarification. We address each point below in the order presented.

Point 1 — Robustness and sensitivity of the epidemic threshold criterion

“The definition of the epidemic threshold, while now clearly specified, remains based on a heuristic transition criterion, and the manuscript would be strengthened by a brief discussion of its robustness or sensitivity.”

We agree that this point deserves explicit discussion. The epidemic threshold is estimated via the susceptibility-peak criterion χ(β) = n·Var(ρ)/(ρ¯ + ε), which is a widely used operational estimator in the computational epidemiology literature (Chakrabarti et al., 2008; Pastor-Satorras et al., 2015). As already noted in the manuscript (Section 4.3), N_mc = 20 Monte Carlo replicates and N_β = 25 grid points were used. The robustness analysis over μ ∈ {0.3, 0.5, 0.7} (Section 5.5, Figure 5b) implicitly assesses sensitivity to this estimator, since varying μ shifts the location of the threshold along the β axis. We have added a concise paragraph in Section 4.3 acknowledging the heuristic nature of the susceptibility-peak estimator and its known sensitivity in specific regimes (e.g., strongly periodic networks), referencing the PRIMARY SCHOOL case discussed in Section 7.4 as a concrete illustration. We also note that a multi-criteria comparison (persistence time, final epidemic size, branching factor) is an important direction for future work, as now stated explicitly in the Limitations paragraph.

Manuscript change: Added ∼3 sentences to Section 4.3 (after Eq. 1) discussing the robustness of the susceptibility-peak estimator and its sensitivity in strongly periodic networks, with a forward reference to Section 7.4 (PRIMARY SCHOOL). Added one sentence to Section 8 (Limitations) on multi-criteria threshold estimation as future work.

Point 2 — Use of τ_QMF as an input feature versus benchmark

“The use of τ_QMF as an input feature, while now acknowledged, continues to raise some interpretational questions in relation to the benchmarking against QMF itself, and this point could be clarified further.”

We thank the reviewer for pressing on this point. The potential for circular reasoning is a legitimate concern and deserves a clearer explanation. The key clarification is as follows: τ_QMF = 1/ρ(Ā‾) is used as one of the input features to the physics-guided neural network because it encodes a theoretically motivated baseline (the mean-field approximation), not because it already provides an accurate estimate of λ_c. The benchmarking comparison is between (i) the raw QMF estimate τ_QMF used as a standalone predictor and (ii) our neural network that takes τ_QMF among other spectral features as input and outputs a corrected estimate. The improvement thus measures how much the network learns to correct the systematic bias of the QMF approximation using additional structural information. This is analogous to residual learning in physics-informed machine learning: the model learns the deviation from a known imperfect baseline. We have added an explicit clarifying note in Section 3 (Feature Design) to make this distinction unambiguous for the reader.

Manuscript change: Added a clarifying note (∼4 sentences) in Section 3 explaining that τ_QMF serves as a physics-motivated input feature encoding the mean-field baseline, and that the benchmarking evaluates the network’s capacity to correct this baseline—not a circular comparison. Analogy to residual learning made explicit.

Point 3 — Scope of validity of the physics-guided constraints

“The constraints introduced in the model, although useful as regularizers, remain only loosely connected to formal theoretical derivations, and their scope of validity could be discussed more explicitly.”

We agree with the reviewer that the theoretical grounding of the physics-guided constraints merits more explicit treatment. The constraints encode two well-established properties of the epidemic threshold: (C1) monotone decrease with network connectivity (captured by the positivity constraint on the spectral-radius coefficient), and (C2) positivity of predictions (guaranteed by log-space parameterisation). Both are direct consequences of the QMF approximation λ_c ∼ μ/ρ(Ā‾) under the assumptions of the mean-field model (homogeneous recovery, Markovian SIS dynamics). We now explicitly state in Section 3 that these constraints are valid within the regime of applicability of the QMF approximation—namely, networks with moderate temporal heterogeneity (σ²_λ/λ‾²₁ < 0.5, as defined in Section 6)—and may lose theoretical motivation for strongly heterogeneous networks (e.g., activity-driven configurations), where they act primarily as regularizers ensuring numerical stability and output positivity.

Manuscript change: Added ∼3 sentences to Section 3 (Physics-Guided Constraints) clarifying: (a) the theoretical basis of each constraint within the QMF regime; (b) the regime of validity (σ²/λ‾² < 0.5); (c) their role as regularizers outside this regime. Cross-reference to Theorem 6.1 and Remark 6.2 added.

Point 4 — Scope of applicability claims

“The study is still largely restricted to synthetic networks, and although this limitation is recognized, the claims regarding applicability should remain cautious in the absence of validation on empirical data.”

We fully agree. While Section 7 provides empirical validation on four SocioPatterns real-world contact networks, the neural network itself was trained exclusively on synthetic data, and the empirical results are presented as out-of-distribution generalization tests rather than a comprehensive validation. We have revisited the Abstract, Introduction (end of Section 1.2), and Discussion (Section 8) to replace unqualified applicability claims with appropriately hedged formulations. Specifically, phrases of the form “directly applicable to data from hospitals, schools, and conferences” have been revised to “potentially applicable, subject to empirical validation on data from [context]”, and the in-regime/out-of-regime distinction is now referenced in these contexts.

Manuscript change: Revised 3 sentences in the Abstract, Section 1.2, and Section 8 to replace unqualified applicability claims with hedged formulations referencing the in-regime condition (σ²/λ‾² < 0.5) and the need for further empirical validation. No structural or methodological changes.

Summary of Changes

The table below summarises all modifications made to the manuscript in this revision round.

# Reviewer concern Change made Location

1 Robustness of threshold criterion Added ∼3 sentences on sensitivity of susceptibility-peak estimator; future work on multi-criteria estimation Sec. 4.3, Sec. 8

2 τ_QMF as feature vs benchmark Added clarification: τ_QMF encodes the mean-field baseline; network learns to correct it (residual learning analogy) Sec. 3

3 Scope of physics-guided constraints Added theoretical basis, regime of validity (σ²/λ‾² < 0.5), and regularizer role outside regime Sec. 3, cross-ref Thm 6.1

4 Applicability claims to empirical data Revised 3 sentences: unqualified claims → hedged formulations referencing in-regime condition Abstract, Sec. 1.2, Sec. 8

Closing Statement

We believe that the revised manuscript fully addresses the minor concerns raised in this round. The core contributions—the physics-guided neural network architecture, the rigorous evaluation protocol (LOO-CV, bootstrap CIs, ablation study), and the public code/data availability—remain unchanged. We are confident that the manuscript is now ready for publication in PLOS ONE and hope that the Editor and Reviewer #2 will find the revised version satisfactory.

We thank the editorial team for their continued support of this work.

Sincerely,

Dr. Etienne Kouokam

Université de Yaoundé I / IRD-UMMISCO, Sorbonne Université

etienne.kouokam@facsciences-uy1.cm

Attachment Submitted filename: Response_to_Reviewers_PONE_R3.docx

# Editor report: Decision Letter 2

**DOI:** 10.1371/journal.pone.0344827.r005

**Authors:** Americo Cunha

28 May 2026

A Hybrid AI-Mathematical Approach for Epidemic Threshold Prediction in Metapopulation Networks: .Integrating Physics-Guided Neural Networks with Spectral Graph Theory

PONE-D-26-09719R2

Dear Dr. Kouokam,

We’re pleased to inform you that your manuscript has been judged scientifically suitable for publication and will be formally accepted for publication once it meets all outstanding technical requirements.

Within one week, you’ll receive an e-mail detailing the required amendments. When these have been addressed, you’ll receive a formal acceptance letter and your manuscript will be scheduled for publication.

An invoice will be generated when your article is formally accepted. Please note, if your institution has a publishing partnership with PLOS and your article meets the relevant criteria, all or part of your publication costs will be covered. Please make sure your user information is up-to-date by logging into Editorial Manager at Editorial Manager® and clicking the ‘Update My Information' link at the top of the page. For questions related to billing, please contact billing support.

If your institution or institutions have a press office, please notify them about your upcoming paper to help maximize its impact. If they’ll be preparing press materials, please inform our press team as soon as possible -- no later than 48 hours after receiving the formal acceptance. Your manuscript will remain under strict press embargo until 2 pm Eastern Time on the date of publication. For more information, please contact onepress@plos.org.

Kind regards,

Americo Cunha Jr

Academic Editor

PLOS One

Additional Editor Comments (optional):

The revised manuscript has undergone substantial improvement and now satisfactorily addresses the major concerns raised during the review process. The methodological foundations of the study have been significantly strengthened, particularly through the clearer definition of the epidemic threshold ground truth, the addition of Monte Carlo–based uncertainty quantification, and the clarification of the learning problem formulation to avoid ambiguity and potential leakage. The repositioning of the approach as a physics-guided rather than physics-informed framework results in a more accurate and scientifically balanced presentation of the work.

The experimental evaluation has also been considerably expanded and strengthened through the inclusion of additional baselines, repeated cross-validation, statistical assessment, ablation studies, interpretability analysis, and public availability of code and data, substantially improving transparency and reproducibility. The remaining limitations of the study—particularly regarding the heuristic nature of the threshold estimator, the use of synthetic networks, and the scope of applicability of the constraints—are now explicitly acknowledged and discussed appropriately.

Overall, the manuscript is now methodologically sound, substantially more transparent, and scientifically balanced. The remaining issues are minor, do not compromise the validity of the work, and are acceptable within the scope and editorial standards of PLOS ONE.

Reviewers' comments:

# Editor report: Acceptance letter

**DOI:** 10.1371/journal.pone.0344827.r006

**Authors:** Americo Cunha

PONE-D-26-09719R2

PLOS One

Dear Dr. Kouokam,

I'm pleased to inform you that your manuscript has been deemed suitable for publication in PLOS One. Congratulations! Your manuscript is now being handed over to our production team.

At this stage, our production department will prepare your paper for publication. This includes ensuring the following:

* All references, tables, and figures are properly cited

* All relevant supporting information is included in the manuscript submission,

* There are no issues that prevent the paper from being properly typeset

You will receive further instructions from the production team, including instructions on how to review your proof when it is ready. Please keep in mind that we are working through a large volume of accepted articles, so please give us a few days to review your paper and let you know the next and final steps.

Lastly, if your institution or institutions have a press office, please let them know about your upcoming paper now to help maximize its impact. If they'll be preparing press materials, please inform our press team within the next 48 hours. Your manuscript will remain under strict press embargo until 2 pm Eastern Time on the date of publication. For more information, please contact onepress@plos.org.

You will receive an invoice from PLOS for your publication fee after your manuscript has reached the completed accept phase. If you receive an email requesting payment before acceptance or for any other service, this may be a phishing scheme. Learn how to identify phishing emails and protect your accounts at https://explore.plos.org/phishing.

If we can help with anything else, please email us at customercare@plos.org.

Thank you for submitting your work to PLOS ONE and supporting open access.

Kind regards,

PLOS ONE Editorial Office Staff

on behalf of

Dr. Americo Cunha Jr

Academic Editor

PLOS One
